# Comparative Phylogeographic Analyses Illustrate the Complex Evolutionary History of Threatened Cloud Forests of Northern Mesoamerica

**DOI:** 10.1371/journal.pone.0056283

**Published:** 2013-02-07

**Authors:** Juan Francisco Ornelas, Victoria Sosa, Douglas E. Soltis, Juan M. Daza, Clementina González, Pamela S. Soltis, Carla Gutiérrez-Rodríguez, Alejandro Espinosa de los Monteros, Todd A. Castoe, Charles Bell, Eduardo Ruiz-Sanchez

**Affiliations:** 1 Departamento de Biología Evolutiva, Instituto de Ecología, AC, Xalapa, Veracruz, Mexico; 2 Department of Biology, University of Florida, Gainesville, Florida, United States of America; 3 Instituto de Biología, Universidad de Antioquia, Medellín, Antioquia, Colombia; 4 Florida Museum of Natural History, Gainesville, Florida, United States of America; 5 Department of Biology, University of Texas Arlington, Arlington, Texas, United States of America; 6 Department of Biological Sciences, University of New Orleans, New Orleans, Louisiana, United States of America; University of York, United Kingdom

## Abstract

Comparative phylogeography can elucidate the influence of historical events on current patterns of biodiversity and can identify patterns of co-vicariance among unrelated taxa that span the same geographic areas. Here we analyze temporal and spatial divergence patterns of cloud forest plant and animal species and relate them to the evolutionary history of naturally fragmented cloud forests–among the most threatened vegetation types in northern Mesoamerica. We used comparative phylogeographic analyses to identify patterns of co-vicariance in taxa that share geographic ranges across cloud forest habitats and to elucidate the influence of historical events on current patterns of biodiversity. We document temporal and spatial genetic divergence of 15 species (including seed plants, birds and rodents), and relate them to the evolutionary history of the naturally fragmented cloud forests. We used fossil-calibrated genealogies, coalescent-based divergence time inference, and estimates of gene flow to assess the permeability of putative barriers to gene flow. We also used the hierarchical Approximate Bayesian Computation (HABC) method implemented in the program msBayes to test simultaneous versus non-simultaneous divergence of the cloud forest lineages. Our results show shared phylogeographic breaks that correspond to the Isthmus of Tehuantepec, Los Tuxtlas, and the Chiapas Central Depression, with the Isthmus representing the most frequently shared break among taxa. However, dating analyses suggest that the phylogeographic breaks corresponding to the Isthmus occurred at different times in different taxa. Current divergence patterns are therefore consistent with the hypothesis of broad vicariance across the Isthmus of Tehuantepec derived from different mechanisms operating at different times. This study, coupled with existing data on divergence cloud forest species, indicates that the evolutionary history of contemporary cloud forest lineages is complex and often lineage-specific, and thus difficult to capture in a simple conservation strategy.

## Introduction

**Figure 1:**
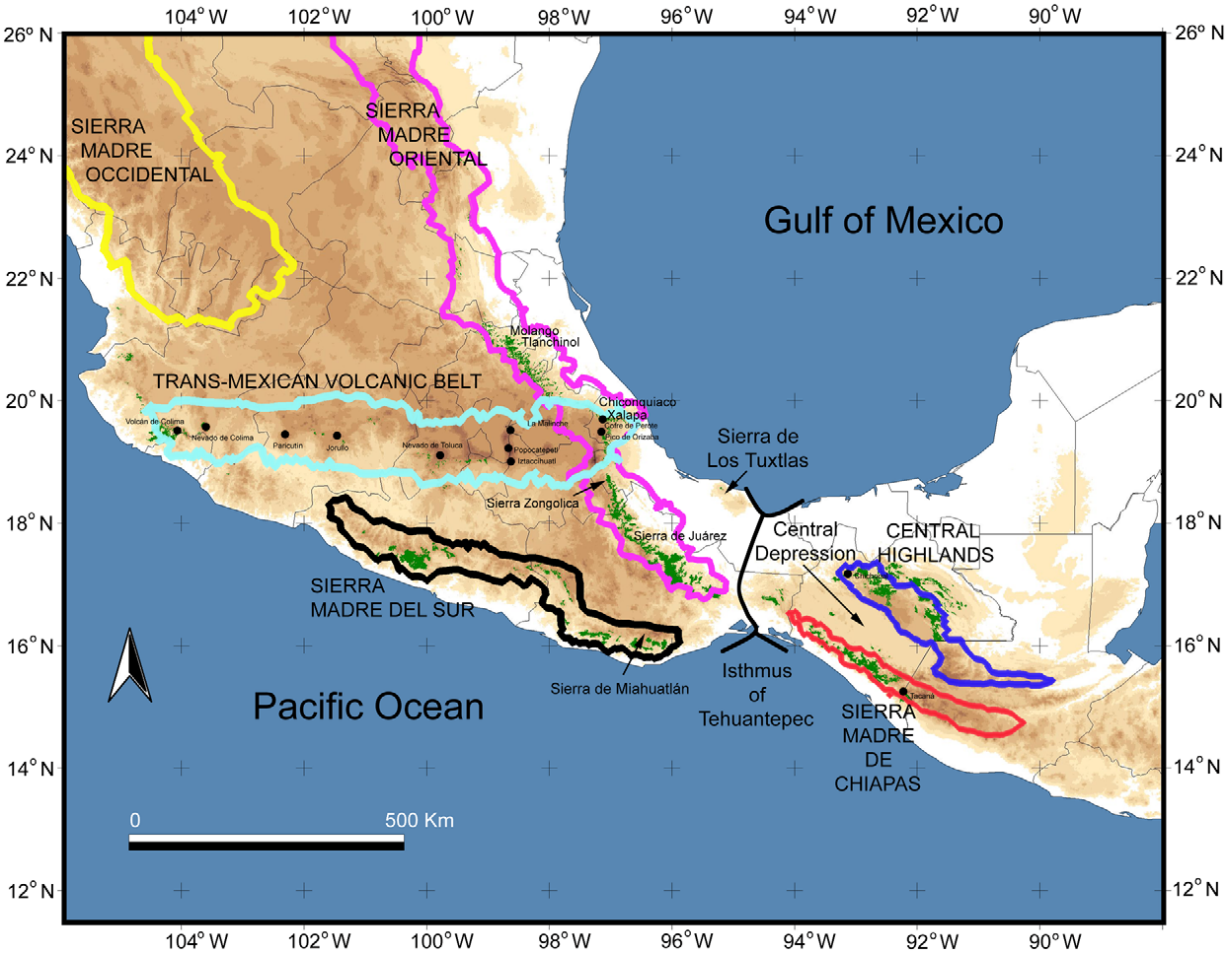
Geographical regions of Mexico and current natural range of cloud forests (indicated by green shading) in eastern Mexico overlaid on a relief map. The contour colored lines correspond to Sierra Madre Occidental (yellow), Sierra Madre Oriental (pink), Trans-Mexican Volcanic Belt (light blue), Sierra Madre del Sur (black), Sierra Madre de Chiapas (red), and the Central Highlands of Chiapas (dark blue). Note the geographic disjunction at the Isthmus of Tehuantepec and isolation of Sierra de Los Tuxtlas.

Cloud forest is among the most threatened types of vegetation in northern Mesoamerica. This ecological community currently occupies less than 1% of the total area in Mesoamerica, and 50% of the original (pre-European) cloud forest area has been lost and replaced by other vegetation types [1], [2]. In northern Mesoamerica, from southern Tamaulipas in eastern Mexico to the Guatemalan highlands, this forest type exists as small isolated fragments restricted to ravines and patches along mountain slopes from 600 to 2000 m above sea level [3]. Cloud forests exhibit a large geographical disjunction at the Isthmus of Tehuantepec in Mexico, separating forest patches along the Sierra Madre Oriental and Sierra de Los Tuxtlas to the north from those in Chiapas and the Guatemalan highlands to the south (Figure 1). Throughout its geographic distribution, cloud forest habitat is influenced by fog during the winter dry season, and precipitation ranging from 1000 to 3000 mm of annual precipitation, and average daily temperatures from 12 to 23 °C and an annual mean around 18 °C [4]. These conditions favor the development of exuberant vegetation, including large tree ferns, a great variety of epiphytes and vines, and a mixed canopy of temperate deciduous trees and tropical broadleaved-evergreen trees [5]. Cloud forest also recognized for its high endemism of epiphytes, has the highest biotic diversity per unit area in Mexico with cloud forests accounting for 10% of all Mexican flora (2500 vascular plant species) and 12% of the terrestrial vertebrates [2]–[4], [6]–[8].

Although this biome harbors numerous endemic endangered species (e.g., the Resplendent Quetzal, *Pharomachrus mocinno*, the Horned Guan, *Oreophasis derbianus*, and the Yolosúchil *Magnolia* [*Talauma*] *mexicana*), it is being lost at an alarming rate due to human disturbance [9], and our understanding of patterns of regional divergence and endemic genetic diversity is limited. Previous phylogeographic studies of the Mesoamerican cloud forest biodiversity have emphasized species that have migrated south from North America [10]–[13]. Few studies, however, have been conducted on representatives of the Mesoamerican biota hypothesized to have migrated from South America [14], [15]. The phylogeographic patterns of temperate tree species with North American origins have been mainly attributed to isolation and climate changes during Pleistocene glaciations that promoted the expansion, fragmentation, and divergence of populations [11], [16]. In *Fagus grandifolia* and *Liquidambar styraciflua*, two widespread tree species that occur in both the eastern USA and eastern Mexico, all individuals in the Mexican populations surveyed shared widespread cpDNA haplotypes; this finding suggests recent gene flow between populations [12], [13]. In contrast, divergence patterns of plant species that colonized northern Mesoamerica from South America are more likely to have been shaped by a greater diversity of historical processes occurring at different times, such as Quaternary glaciations and pre-Quaternary palaeogeographical dynamics [14], [15].

Similar to divergence patterns in plants, phylogeographic investigations of cloud forest bird species have identified two discrete diversification events, estimated as occurring in the Pleistocene or late Pliocene, separating populations that occur in cloud forests on either side of the Isthmus of Tehuantepec and currently isolated from one another by the intervening valley [17]–[20]. Fluctuations in cloud forests induced by climatic cycles and a late Pliocene seaway at the isthmus have been implicated in subdividing the distribution of this bird fauna spanning the isthmus [18]. The divergence times estimated for both plants and birds strongly support the model of a highly constrained temporal window at the end of the Pliocene when a majority of bird diversification events occurred; a second period of divergence also apparently occurred earlier in the Miocene across the isthmus, but mainly for plants [14].

Here we use a comparative phylogeographic approach [21], [22] to examine patterns of genetic divergence among co-distributed plant and animal cloud forests species. Using DNA sequence data, we test the hypothesis of simultaneous intraspecific divergence across 15 species whose distributions span the cloud forest of northern Mesoamerica (details of collection sites and DNA sequences available in Table S1). We used phylogenetic trees that were time-calibrated using fossils, and coalescent-based estimates of divergence times and gene flow to test whether these species share the same phylogeographic breaks. In addition, we performed analysis on the multiple species data set to test simultaneous divergence versus non-simultaneous divergence of cloud forest species. Specifically, we asked the following questions: (1) Is there evidence that temporal and geographical patterns of divergence are shared among multiple co-distributed cloud forest species? If so, is there evidence for what the underlying geological or climatic causes might be? (2) Did cloud forest species diverge at the Isthmus of Tehuantepec (or at other phylogeographic breaks) primarily during the late Pliocene and Pleistocene? (3) Is there evidence of more ancient divergence events observed during the late Miocene, when aridity increased in most of Mexico and dramatic geological events and volcanism took place? (4) Is there phylogeographic signal in our data set that can be used to formulate an explicit model of cloud forest speciation during either glacial and/or interglacial periods?

To address these questions, we use DNA sequence data for 15 species in the analyses: new data are presented here for 10 species (1233 individuals, *n* = 67–193 per species) and we employed published data for an additional five species (231 individuals, *n* = 18–104 per species). We included five plant species (one gymnosperm and four angiosperms) including the trees *Podocarpus matudae* (Podocarpaceae) and *Liquidambar styraciflua* (Altingiaceae), the shrub *Palicourea padifolia* (Rubiaceae), the herb *Moussonia deppeana* (Gesneriaceae), and the epiphytic *Rhipsalis baccifera* (Cactaceae); three rodent species (*Peromyscus aztecus*, *Reithrodontomys sumichrasti*, *Habromys lophurus*). The seven remaining species include three hummingbird species *Amazilia cyanocephala*, *Campylopterus curvipennis* and *Lampornis amethystinus* (Trochilidae), a woodcreeper *Lepidocolaptes affinis* (Furnariidae), and three passerine species, *Chlorospingus ophthalmicus* and *Buarremon brunneinucha* (Emberizidae) and *Basileuterus belli* (Parulidae). Included in our analyses are eight species with North American origins (*P. matudae*, *L. styraciflua*, *M. deppeana*, *B. brunneinucha*, *C. ophthalmicus*, and the three rodent species), five species with South American origins (*P. padifolia*, *R. baccifera*, *C. curvipennis*, *A. cyanocephala*, *L. affinis*), and two species with Central American origins (*L. amethystinus*, *B. belli*). The 15 taxa were also chosen because they are thought to be cloud forest endemics whose distributions span northern Mesoamerica and closely follow the highly fragmented forest; all of the birds are considered to be cloud forest residents. Some of the targeted species ecologically interact with one another; *P. padifolia* and *M. deppeana* are mainly pollinated by the hummingbird species (*C. curvipennis*, *A. cyanocephala*, and *L. amethystinus*, respectively) and seeds of *R. baccifera* are dispersed by *C. ophthalmicus* [15], [19], [20]. Our *a priori* expectation was that such ecologically linked species might share a common biogeographic history. Here we compare patterns of divergence within and among these 15 species, and test the hypothesis of shared spatio-temporal vicariance across taxa having led to patterns of cloud forest biodiversity.

## Materials and Methods

### Ethics statement

We obtained collecting permits to conduct this work from the Secretaría de Medio Ambiente y Recursos Naturales, Instituto Nacional de Ecología, Dirección General de Vida Silvestre (permit numbers: DOO/02/3269, INE SGPA/DGVS/02473/06, INE SGPA/DGVS/02038/07, INE SGPA/DGVS/01568/08, INE SGPA/DGVS/02517/09) and the Guatemalan Government through the Universidad del Valle de Guatemala herbarium (UVAL) collecting permits granted to José Juan Vega (Universidad San Carlos de Guatemala) and Ana Lu MacVean (Universidad del Valle de Guatemala). All necessary permits were obtained for the described field studies. Leaf tissue samples were obtained from the plant species reported here with no further manipulation. Birds were captured in mist nets and the two outer tail rectrices were collected for subsequent genetic analysis before they were released. All procedures with birds were carried out in accordance with the Guidelines for the Use of Wild Birds in Research proposed by the North American Ornithological Council and approved by the Consejo Nacional de Ciencia y Tecnología (CONACyT, # 61710, 25888), Instituto de Ecología, AC (INECOL, # CPE/2006/122), Universidad Nacional Autónoma de México (UNAM) Graduate Studies Committee (Doctorado en Ciencias Biomédicas), and the Instituto de Ecología (INECOL) Graduate Studies Committee (Doctorado en Biodiversidad y Sistemática). The field studies did not involve endangered or protected species.

### Genetic data and analyses

DNA samples were primarily obtained through our own collecting efforts between 2005–2010 along the Sierra Madre Oriental, Sierra de Los Tuxtlas, Sierra de Miahuatlán, Sierra de Juárez, Sierra de Manantlán, and in the highlands of Chiapas and Guatemala (Figure 1; Table S1). DNA data sets were obtained as part of ongoing projects investigating the evolutionary history of cloud forests in eastern Mexico. For the bird species, sequence data from the mitochondrial NADH dehydrogenase subunit 2 gene (ND2) and subunit 5 gene (ND5), cytochrome *b* (CYTB), control region (CR), and ATPase 6 and 8 (ATP6, ATP8) were obtained from DNA extracted from tail feathers or tissue samples using standard molecular laboratory protocols, which are fully described elsewhere [17], [19], [20]. For the plant taxa, sequence data from the nuclear ribosomal DNA (nrDNA) internal transcribed spacer (ITS) and plastid *rpl*32-*trn*L, *psb*A-*trn*H, *trn*S-*trn*G, and *trn*L-*trn*F intergeneric spacers were obtained from silica-dried leaf material also using standard protocols (e.g., [14], [15]). New DNA sequences reported in this paper have been deposited at GenBank with the accession numbers specified in Table S1. ND2 and CYTB sequence data of several rodents (*P. aztecus* complex, 18 individuals; *R. sumichrasti*, 30 individuals; *H. lophurus* complex, 31 individuals) and ATP6, ATP8, ND2, and CYTB of additional birds (*B. brunneinucha*, 48 individuals; *L. affinis*, 104 individuals) from published studies [23]–[27] were also included. The DNA sequences from published sources were obtained from GenBank with the accession numbers also specified in Table S1. Methods for DNA preparation, PCR amplification, and sequence generation are given elsewhere [14], [15], [17], [19], [20], [23]–[27].

Intraspecific phylogenetic relationships among haplotypes were reconstructed using Bayesian inference in MrBayes v. 3.12 [28]. The Bayesian analyses were performed separately on concatenated datasets, treating all individuals with the same haplotype as a single sample for analyses. We implemented jModeltest v. 0.1.1 [29] to establish which substitution models best fit each dataset (Table S2). We allowed four incrementally heated Markov chains to proceed for 10 million generations, sampling every 1000 generations. Bayesian posterior probability values were estimated from the sampled trees remaining after 1000 burn-in samples were discarded [28]. Statistical parsimony haplotype networks were obtained with the program TCS v. 1.2.1 [30] using the 95% connection probability limit. Some ambiguities (loops) were detected in the networks, and were broken according to three criteria (frequency, topology and geography), as proposed by Pfenninger and Posada [31]. Lineage splits highly supported (*PP*>0.9) by Bayesian analyses, haplotype networks and/or previously published phylogenies [23]–[27] were considered for identifying geographical barriers. We used Arlequin v. 3.1 [32] to calculate sequence divergence using the Tamura-Nei model of sequence evolution and corrected distances within-(*Dx, Dy*) and between-populations (*Dxy*) for the observed vicariant events.

### Divergence time estimation

To relate genetic differentiation to historical events, we estimated time-to-most-recent-common-ancestor (tMRCA) and the confidence interval for each taxon using the program BEAST v. 1.5.4 [33]. Details on molecular markers, substitution models, substitution rates, temporal calibrations of the trees, and taxon sampling can be found in Table S2. In all cases we used an uncorrelated lognormal relaxed clock model and a coalescent model assuming population constant size, except in two cases (*P. matudae* and *M. deppeana*) for which we did additional analyses using the Yule speciation model (see Text S1). For divergence time estimations the Markov chain Monte Carlo (MCMC) analyses of each species consisted of one to four independent runs each of 10 or 20 million generations, with samples retained every 1000 generations. Results were visualized in Tracer v. 1.5 (http://tree.bio.ed.ac.uk/software/tracer/) to confirm mixing, burn-in, and adequate effective sample sizes (ESS>200 for each estimated parameter). After discarding the first 10% of the trees as burn-in, the trees and parameter estimates from the independent runs were combined using LogCombiner v. 1.5.2 [33]. The parameter values of the remaining samples from the posterior distribution were summarized on the maximum clade credibility tree using TreeAnnotator v. 1.5.2 [33], with posterior probability limit set to 0.5 and mean node heights summarized.

The fossil record for all of the systems included in our study is meager, and no reliable fossil evidence is presently available in most cases. Thus, we conducted the dating analyses with fossil calibration points when such data were available. Otherwise, substitution rates, and combinations of fossil calibration points and dates estimated from other papers (secondary calibrations) were used to obtain dates for splits of particular interest. Details of the nucleotide evolutionary models used, fossil and rate calibrations, topological constraints, prior distribution and parameter values used for analyses in BEAUti v. 1.5.4 [33], as well as taxon sampling details, can be found in Text S1. Lastly, we used the program TreeStat v. 1.2 [34] to summarize the Markov chain results for posterior divergence date estimates, and used an R script to create posterior density plots for nodes of interest.

### Gene flow

Gene flow between populations on either side of geographic barriers (e.g., Isthmus of Tehuantepec) was estimated using IMa [35] to estimate confidence intervals for migration rates; these estimates were used as priors for tests of simultaneous diversification using Approximate Bayesian Computation (ABC) methods. For IMa analyses, we performed preliminary analyses with few steps and low burn-in periods to specify the appropriate priors for each analysis. When parameter curves were adjusted we reran the analyses with burn-in periods of 300,000–60,000,000 steps, and with 1,000,000–56,000,000 steps in the chain following burn-in. Convergence was assumed when effective sample sizes (ESSs) were greater than 50 for all parameters [35]. We obtained the lower and higher probability density estimates of migration rates between populations (*m_1_, m_2_*), which were converted to the effective number of immigrants per generation by using estimates of theta.

### Tests of simultaneous diversification

If the same biogeographic barrier had similarly impacted different lineages (i.e. causing vicariance), it would be expected that different lineages would share similar divergence times across the barrier. However, incongruence in divergence times between taxon pairs does not rule out the possibility of simultaneous divergence. Even in a vicariant event, differences in times of divergence can also be due to variance in the coalescent process; in other words, this difference in divergence time estimated may represent a discrepancy between gene divergence time and population time divergence, which is related to the demography of each particular species [36]. Here, to address this issue, a hierarchical Approximate Bayesian Computation (HABC) method implemented in the program msBayes [37], [38] was employed to evaluate competing hypotheses: simultaneous divergence versus non-simultaneous divergence. Our hierarchical Bayesian approach aims to estimate hyper-parameters such as the number of distinct divergence times among lineages, while accounting for variation in demographic parameters (e.g., effective population sizes, mutation and migration rates) within lineages conditioned on the hyper-parameters. By doing so, we were able to test the predictions under two different biogeographic scenarios: single vs. multiple divergence events. The HABC method reasonably distinguishes simultaneous isolation from temporal incongruence in the divergence of co-distributed population pairs, even with sparse sampling of individuals, and has been shown to be effective over a range of conditions with a single locus [37]–[41].

msBayes analyses involved three stages. First, we estimated summary statistics from the observed sequences. Second, we simulated a large number of data sets (1,000,000) under the scenario of multiple divergence times (hyper-parameter) using prior distribution for the demographic sub-parameters. We allowed the maximum value for Theta to be estimated as part of the analyses. The obtained value was 0.15816, which is appropriate for mtDNA (M. Hickerson, pers. com.). The prior for the tau parameter was set to 3 based on the divergence times obtained from the BEAST analyses. Finally, the prior for the maximum possible number of divergence events (Ψ) was set to be equal to the number of lineage pairs spanning each barrier. Although the newest version of msBayes allows for multi-locus data [42], our datasets include a single locus for each pair of species. Thus, we inferred co-divergence among taxon pairs by ruling out differences in coalescent times using a single locus (i.e. mtDNA).

Because gene flow after divergence can affect coalescent processes, we first estimated migration rates across divergent populations of each species using the Isolation-with-Migration model implemented in the program IMa (described above). The mean migration rate per biogeographic barrier was incorporated as prior information in the simulation stage of msBayes. For each of the 1,000,000 data sets we obtained the summary statistics as we did for the observed sequences. Lastly, we randomly drew 2,000 samples from the simulated data sets and used the acceptance/rejection algorithm to approximate the posterior distribution for the hyper parameters (e.g., Ψ). We used Ω estimates to evaluate the relative support for each hypothesis, based on recent evidence that Ω estimates are more reliable indicators than Ψ estimates [43].

## Results

### Phylogeny and divergence time estimation

The phylogenies of all 15 species recovered varying levels of sequence divergence between populations separated by a geographic barrier, ranging from 0.118% in *R. baccifera* populations separated by the Isthmus of Tehuantepec, to 7–13% in rodent populations separated by this barrier, with intermediate values between populations of bird species (Table S3). Lineage splits highly supported (*PP*>0.9) by the Bayesian analyses and/or suggested by haplotype networks were used to identify phylogeographic barriers. Divergence time estimates (in million of years before present, Ma) within each of the species derived from the BEAST analyses are summarized in Table 1. Effective sample sizes (ESSs) were over 200 for nearly all parameters including time-to-most-recent-common-ancestor (tMRCA). Geographically shared divergence by more than two species was identified in four geographic barriers (Table 1): at the Isthmus of Tehuantepec (east and west of the isthmus), Los Tuxtlas (isolated from the Sierra Madre Oriental), Chiapas populations (separated by the Central Depression), and the Chiapas and Los Tuxtlas populations separated from populations west of the Isthmus of Tehuantepec (Isthmus 2). Phylogeographic breaks and divergence times were not, however, the same across species (Table 1).

**Table 1:** Divergence time estimates (in Mya) for 15 cloud forest taxa that span the geographic barriers identified in this study for each of the taxa derived from the BEAST analyses.

Taxon	Biogeographic affinity	Isthmus of Tehuantepec	Los Tuxtlas	Isthmus 2	Chiapas Central Depression
**Plants**					
*Podocarpus matudae*	NA			25.34 (40.11–8.90)	
*Liquidambar styraciflua*	NA		1.76 (3.41–0.11)		
*Palicourea padifolia*	SA	8.78 (17.05–2.39)			
*Moussonia deppeana*	NA			3.90 (4.83–1.32)	
*Rhipsalis baccifera*	SA	4.20 (5.46–2.49)			
**Birds**					
*Campylopterus curvipennis*	SA	0.88 (1.73–0.29)	0.40 (0.76–0.14)		
*Amazilia cyanocephala*	SA	0.27 (0.51–0.09)			
*Lampornis amethystinus*	CA	1.07 (1.46–0.71)			0.66 (0.91–0.42)
*Lepidocolaptes affinis*	SA	1.55 (3.10–0.45)			
*Buarremon brunneinucha*	NA		1.37 (1.80–0.96)		
*Basileuterus belli*	CA	1.15 (1.71–0.67)	0.16 (0.30–0.02)		0.43 (0.71–0.16)
*Chlorospingus ophthalmicus*	NA		1.92 (3.21–0.82)	3.21 (4.60–1.80)	
**Rodents**					
*Habromys “lophurus”*	NA	1.78 (3.05–0.72)			
*Reithrodontomys sumichrasti*	NA	2.86 (3.78–2.04)			
*Peromyscus “aztecus”*	NA	2.01 (3.18–1.44)			

Ranges correspond to 95% highest posterior density (HPD).

Biogeographic affinity: NA = North America, SA = South America, CA = Central America.

Geographic barrier: Isthmus of Tehuantepec (east and west of the isthmus), Los Tuxtlas (isolated from the Sierra Madre Oriental), Chiapas Central Depression (populations in Chiapas separated by the Central Depression), Isthmus 2 (the Chiapas and Los Tuxtlas populations separated from populations west of the Isthmus of Tehuantepec).

**Figure 2:**
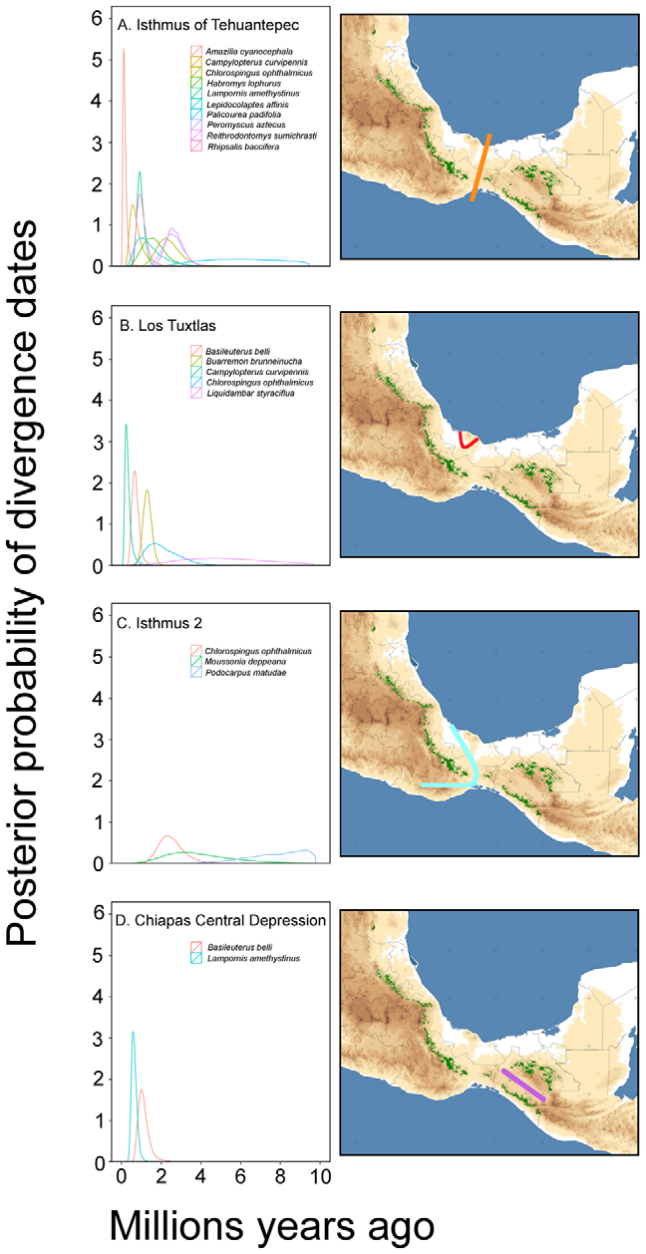
Posterior density plots of divergence times for cloud forest species across four phylogeographic barriers detected. (A) Isthmus of Tehuantepec (east and west of the isthmus). (B) Los Tuxtlas (isolated from the Sierra Madre Oriental). (C) Isthmus 2 (the Chiapas and the Los Tuxtlas populations separated from populations west of the Isthmus of Tehuantepec). (D) Chiapas Central Depression (populations in Chiapas separated by the Central Depression).

There was considerable variation in lineage divergence estimates across lineages tested, and temporal patterns of divergence do not appear to be broadly shared among the co-distributed cloud forest species included in our study (Figure 2). Mean values for the tMRCA ranged from 0.27 Ma for *A. cyanocephala* to 8.78 Ma for *P. padifolia* at the Isthmus of Tehuantepec (Table 1). tMRCA mean values for Los Tuxtlas ranged from 0.16 Ma for *B. belli* to 1.92 Ma for *C. ophthalmicus*, and for the Chiapas populations separated by the Central Depression the tMRCA mean values ranged from 0.43 Ma for *B. belli* to 0.66 Ma for *L. amethystinus*. At Isthmus 2, mean values for the tMRCA were higher and ranged from 3.21 Ma for *C. ophthalmicus* to 25.34 Ma for *P. matudae* (Table 1).

Four additional single divergence events were identified: (1) the split between *R. sumichrasti* populations of the Sierra Madre Oriental from those along the Sierra Madre del Sur (SMO-SMS hereafter) at 1.9 Ma; (2) the split between *B. brunneinucha* populations of the N Oaxacan highlands and Chimalapas region (Oaxaca hereafter) from other populations at 2.3 Ma (95% HPD 2.85–1.87); (3) the split between *M. deppeana* populations of Los Tuxtlas from those in Chiapas and Guatemala (Chiapas-Los Tuxtlas hereafter) Ma at 2.6 (95% HPH 4.22–0.48); and (4) between *M. deppeana* populations of the Sierra de Manantlán and Sierra de Miahuatlán from other populations at 4.83 Ma (95% HPD 7.91–1.32).

### Gene flow

Gene flow estimates between populations on either side of the Isthmus of Tehuantepec were low (<0.004 migrants per generation; m/g), with the exception of *A. cyanocephala* in the east-to-west direction (0.38 m/g) and *R. baccifera* in the west-to-east direction (0.23 m/g) (Table 2). Across the Isthmus 2 barrier, gene flow estimates were higher, ranging from 0.42 m/g in *M. deppeana* to 2.98 m/g in *C. ophthalmicus*. For the other phylogeographic barriers, gene flow estimates were also low, except in *C. ophthalmicus* (0.22 m/g) from Los Tuxtlas to the Sierra Madre Oriental (Table 2). We therefore infer that the unsorted trees we observed for many lineages are primarily the result of incomplete lineage sorting and incomplete coalescence and not due to significant levels of gene flow.

**Table 2:** Estimates of gene flow between populations estimated using IMa.

Geographic barrier	Species	Sample size	m_1_			m_2_		
			mean	HPD90%Lo	HPD90%Hi	mean	HPD90%Lo	HPD90%Hi
Isthmus of Tehuantepec	*Palicourea padifolia*	105/17	0.00311	0.00832	13.53364	0.00008	0.00079	6.39344
	*Rhipsalis baccifera*	134/20	0.00010	0.00016	0.83093	0.23305	0.00019	8.48238
	*Campylopterus curvipennis*	138/21	0.00123	0.00256	0.97837	0.00023	0.00053	1.25425
	*Amazilia cyanocephala*	77/49	0.37889	0.01370	10.90997	0.00252	0.01828	22.63338
	*Lampornis amethystinus*	76/22	0.00137	0.00224	2.03741	0.00129	0.00272	0.89179
	*Lepidocolaptes affinis*	45/25	0.00065	0.00191	1.72650	0.00031	0.00075	1.47128
	*Basileuterus belli*	51/21	0.00081	0.00054	0.84664	0.00016	0.00037	0.86862
	*Habromys “lophurus”*	21/9	0.00126	0.00222	1.70667	0.00094	0.00132	1.93931
	*Peromyscus “aztecus*”	20/3	0.00279	0.01166	1226.92039	0.00000	0.00001	0.03478
	*Reithrodontomys sumichrasti*	23/7	0.00321	0.00624	1.70693	0.00096	0.00114	2.54871
Los Tuxtlas	*Liquidambar styraciflua*	125/9	0.00001	0.00003	5.08616	0.00001	0.00001	12.26216
	*Campylopterus curvipennis*	126/12	0.00099	0.00312	1.46753	0.00011	0.00100	1.96574
	*Basileuterus belli*	13/5	0.00031	0.00038	45.83268	0.00001	0.00002	4.07173
	*Buarremon brunneinucha*	15/2	0.00034	0.00179	118.31991	0.00015	0.00061	142.19836
	*Chlorospingus ophthalmicus*	137/15	0.00834	0.01744	6.82536	0.21650	0.00387	8.97927
Isthmus 2	*Podocarpus matudae*	127/32	0.62980	0.00578	19.89388	0.00048	0.01232	14.07568
	*Moussonia deppeana*	133/60	0.42394	0.01399	2.41157	0.00066	0.00221	0.83061
	*Chlorospingus ophthalmicus*	130/46	2.98087	0.56167	11.562203	0.00625	0.00483	2.64971
Chiapas Central Depression	*Lampornis amethystinus*	16/6	0.00247	0.02435	358.73876	0.00012	0.00041	17.59968
	*Basileuterus belli*	10/11	0.00012	0.00064	15.55927	0.00002	0.00006	3.31018
Chiapas-Los Tuxtlas	*Moussonia deppeana*	12/48	0.00008	0.00071	1.89020	0.00213	0.02316	14.49361
Oaxaca	*Buarremon brunneinucha*	13/33	0.00050	0.00113	2.59232	0.00144	0.00273	1.98903
SMO-SMS	*Reithrodontomys sumichrasti*	15/8	0.00210	0.00394	30.47634	0.00102	0.00111	25.25254

Isthmus of Tehuantepec: Populations on either side of Isthmus of Tehuantepec.

Los Tuxtlas: Los Tuxtlas populations mainly isolated from those along the Sierra Madre Oriental.

Isthmus 2: Populations from Chiapas and Los Tuxtlas separated from populations west of the Isthmus of Tehuantepec (Isthmus 2).

Chiapas Central Depression: Chiapas populations separated by the Central Depression.

Chiapas-Los Tuxtlas: Populations of Los Tuxtlas separated from those in Chiapas and Guatemala.

Oaxaca: Populations of the N Oaxacan highlands and Chimalapas region separated from the rest of the sampled populations.

SMO-SMS: Populations of the Sierra Madre Oriental separated from populations along the Sierra Madre del Sur.

Sample size refers to the number of sequences of each side of the barrier included in the analyses.

### Tests of simultaneous diversification

**Figure 3:**
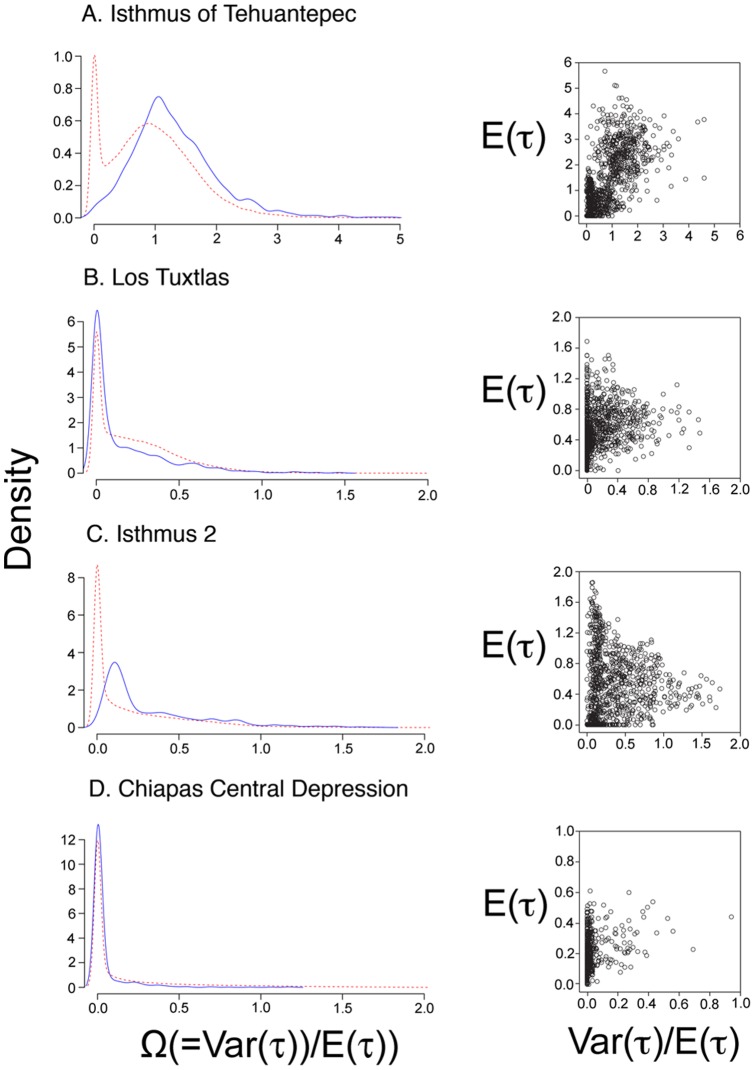
Results of the ABC analyses using msBayes. Posterior probability densities for Ω (left) and the approximate joint posterior estimates of Var(*τ*)/E(*τ*) against E(*τ*) (right) among lineage pairs of plants, birds and rodents spanning. (A) Isthmus of Tehuantepec (*Y* = 10 taxon pairs). (B) Los Tuxtlas (*Y* = 5 taxon pairs). (C) Isthmus 2 (*Y* = 3 taxon pairs). (D) Chiapas Central Depression (*Y* = 2 taxon pairs). Prior (dashed red lines) and posterior (solid blue lines) distributions.

In testing for simultaneous divergence across spatial gaps (four phylogeographic breaks), the msBayes estimates of Omega (Ω) for each of the splits yielded support for non-simultaneous divergence across gaps; the summary of estimated parameters (Ψ and Ω) using msBayes is shown in Table 3. Results indicate that the Chiapas Central Depression, Los Tuxtlas and the Isthmus 2 have likely undergone a single vicariant event (Ψ mode = 1 in all cases). However, the values of Ω, a parameter that measures the incongruence among divergence times along the same phylogeographic barrier, for these three phylogeographic breaks (0.041, 0.169 and 0.332, respectively), all indicate non-simultaneous divergence. Mean values for the tMRCA suggest that splits at these phylogeographic breaks occurred during the late Pleistocene (Figure 2). In contrast, we detected three distinct episodes of divergence (Ψ mode = 3) among the 10 taxa at the Isthmus of Tehuantepec (Figure 3; Table 3), the break for which the Ω value was highest (1.314). Mean values for the tMRCA suggest that *P. padifolia*, *R. baccifera* and the rodents *Peromyscus*, *Reithrodontomys* and *Habromys* split more anciently, and bird taxa *C. curvipennis*, *A. cyanocephala*, *L. amethystinus*, *L. affinis* and *B. belli* experienced more recent divergence events (Figure 2).

**Table 3:** Parameter estimate summaries from the msBayes runs.

Phylogeographic break	*n*	Ψ mode	Ψ mean	Ω mode	Ω mean (95% quantiles)
Isthmus of Tehuantepec	10	3	4.71	1.047	1.314 (0.196–2.996)
Los Tuxtlas	5	1	2.32	0.002	0.169 (0.000–0.843)
Isthmus 2	3	1	1.86	0.107	0.332 (0.028–1.206)
Chiapas Central Depression	2	1	1.24	NA	0.041 (0.000–0.367)

Plants, birds and rodents were combined into single msBayes runs and the disparity in mutation rates was accommodated such that mitochondrial markers evolved 20 times faster than chloroplast and ITS.

n = number of lineage pairs.

Ψ = number of possible divergence times (multinomial logit regression).

Ω = parameter indicating the degree of discordance among divergence times.

## Discussion

### Multiple waves of diversification

Comparative phylogeography studies are capable of revealing cryptic patterns of vicariance, and shared community-wide historical patterns across large regional landscapes [18], [44]–[48]. The HABC method used in this study tests for simultaneous diversification among co-distributed cloud forest taxa, while allowing population histories to vary across taxa [37], [39]. With this approach, we detected non-simultaneous divergence events shared by 2, 3 and 5 cloud forest taxa across the Chiapas Central Depression, Isthmus 2 and Los Tuxtlas barriers, respectively. The timings of the divergence events that are consistent with the tMRCA results using BEAST, however, fall within the Miocene, Pliocene and Pleistocene. At the Isthmus of Tehuantepec, HABC analyses detected several pulses of divergence among 10 cloud forest taxa; a result consistent with the variation in tMRCA estimates, indicating that the genetic divergence observed across different lineage pairs likely occurred variously during different temporal windows. Thus, our comparative data suggests that although some lineages appear to have spatially congruent distributions and genetic breaks, divergences across spatial boundaries are asynchronous among lineages.

Cloud forest taxa with a break at the Isthmus of Tehuantepec did not appear to share the same divergence time. The Isthmus of Tehuantepec, a savannah-like valley approximately 224 m above sea level and some 250 Km wide at its narrowest point, was formed during the Miocene (*c*. 6 Ma) [49]. Geological evidence suggests that from the late Miocene through late Pliocene, an extensive down dropping of the eastern block along the Tehuantepec fault resulted in a massive reduction of the highlands and probably a large marine embayment [49]. The traditional view of a single seaway, however, is insufficient to explain inferences of several episodes of diversification. Although marine incursions are supported by geological data [49], [50], these may not have completely submerged the isthmus valley, and thus may not have resulted in complete breaks in gene flow for some lineages; alternatively, some lineages may have been able to accomplish sufficient gene flow across such seaways (e.g., birds, trees).

A broad-reaching vicariant event during the Pliocene has been suggested as being responsible for the divergence of numerous lineages across the isthmus [15], [18], [19], [45], [51]. Two discrete diversification events across the Isthmus of Tehuantepec were detected for populations of eight bird species occurring in pine-oak forests on either side of the Isthmus and currently isolated from one another by the intervening valley [18]. These two events occurred within the Pleistocene or late Pliocene, suggesting that fluctuations in pine-oak forests induced by climatic cycles and a late Pliocene seaway at the isthmus may have fragmented this montane bird fauna [18]. A congruent temporal divergence across the isthmus in the Pliocene was suggested for *Atropoides* and *Cerrophidion* pitvipers [51]. Similarly, most cladogenic events of several snake lineages across the Isthmus of Tehuantepec occurred between 2.8 and 7.35 Ma [45], supporting a model that includes a highly constrained temporal window of divergence impacting snake lineages at the end of the Pliocene. A previous period of divergence is also detected in the Miocene across the isthmus when a different geological/climatic event may have been responsible for divergence ([14]; this study). Lastly, the temporary isolation during Pleistocene glacial cycles may explain additional patterns of relatively recent diversification in the region ([15], [19], [20]; this study). Altogether, inferred divergence patterns are consistent with a model of vicariance across diverse lineages of plants and animals across the isthmus caused by different mechanisms and operating at different times.

The evolutionary history of contemporary cloud forest lineages reflects a dynamic Mesoamerican geologic landscape. During the late Oligocene to the middle Miocene, the Sierra Madre Occidental uplift and the Trans-Mexican Volcanic Belt (Figure 1) are thought to have changed regional climates and led to establishment of the major biomes of western and central Mexico [52], [53]. The last uplift of the Sierra Madre Occidental occurred between 34 and 15 Ma, whereas the Trans-Mexican Volcanic Belt was formed in several stages in a west-east progression that started in the west *c*. 23 Ma and ended 2.5 Ma [52], [53]. Geological and geochronologic data suggest two major episodes of volcanism in eastern Mexico: the onset of the Trans-Mexican Volcanic Belt that extended to the southeast of Veracruz (Chiconquiaco plateau) placed in the middle Miocene and the initial volcanic activity at Los Tuxtlas [54], [55], and a second eruptive episode in the late Miocene to early Pliocene (7.5–3 Ma) located to the north (Tlanchinol-Molango region) and the east (Chiconquiaco, Palma Sola, Los Tuxtlas) of the previous episode [55]. In late Pliocene and Quaternary, monogenetic volcanism dominated, particularly around Xalapa in central Veracruz [56]. The Chiapan-Guatemalan highlands are largely the result of two distinct tectonic events: the uplift of the extensive northern Central American plateau that took place during the late Miocene to early Pliocene [57], and a younger chain of volcanoes that was formed along the western portion of the Central American plateau in the late Pliocene [58]. The formation of this imposing mountain chain had a significant impact on the biota, resulting in cloud forest conditions on the windward (south) slopes and rain shadow conditions in the interior valleys [59]. These cooler, wetter conditions along the Pacific coast of south-western Guatemala and south-eastern Chiapas are suitable for cloud forest-adapted lineages, which are isolated by from other humid highlands to the south and west by the arid interior basins of the Grijalva and Motagua rivers that drain to the Atlantic–the Central Depression (Figure 1). Although the complex tectonic and volcanic history of the region may have contributed to the high endemicity in northern Mesoamerica, our results and those of previous studies suggest that these more ancient events contributed more to more ancient divergences, and are not directly responsible for the high alpha taxonomic and population genetic diversity observed among highlands, which instead appears to have been generated by more recent events ([13]–[14], [45], [51], [60]; see Table 1). Examples of this include divergences along the Isthmus 2 of *P. matudae*, *M. deppeana* and *C. ophthalmicus*, the divergence of *B. brunneinucha* populations of the N Oaxacan highlands and Chimalapas region from other populations, the divergence of *M. deppeana* populations of the Sierra Madre del Sur (Sierra de Manantlán and Sierra de Miahuatlán) from other populations, and the divergence of *L. styraciflua* populations in the USA from other populations in northern Mesoamerica (see Table 1). Expansions of cloud forest during Pleistocene glacial cycles may have promoted dispersal and periodic bouts of gene flow that could have erased or obscured the genetic signatures of previous historical isolation of populations. In most cases, however, we do find evidence that the climatic complexity that occurred during the Pleistocene also played a role in lineage divergence, particularly for species with Central and South American origins.

Geographic barriers to gene flow within the Miocene and through the Plio-Pleistocene might have changed as a result of much later expansions and arrivals of the cloud forest species during the last Pleistocene glaciations [61], [62]. In northern Mesoamerica, the effects of Pleistocene glaciations were less drastic and more heterogeneous than in the temperate zones because climate is affected not only by latitude but also by altitude and exposure [63]. In Mexico, an average decrease in temperature of 6–8 °C has been reported for the Last Glacial Maximum, together with increased precipitation in some areas [64] and a snowline depression of 1300 m [63]. These changes produced a downward altitudinal migration that broadened distributions of cloud forests that has been documented in paleobotanic records [62]. Undoubtedly, the Isthmus of Tehuantepec has acted as a barrier and a corridor at different times [18], [65]. It is possible that the cloud forests in the Los Tuxtlas region originated more recently and may have become isolated in the higher elevations during the last 40,000 years when the volcanic massif was already formed [66]. Similarly, the arid Chiapas Central Depression was probably more humid during the Pleistocene or early Holocene [67], and thus more conducive to dispersal of cloud forest species between the central highlands of Chiapas and those in the Sierra Madre de Chiapas on the Pacific slope. The recent lineage divergences observed at the Isthmus of Tehuantepec, Los Tuxtlas and the Chiapas Central Depression, suggest that genetic differentiation of species, particularly those with Central and South American origins, is a response to climate-driven cloud forest dynamics. The current distribution of cloud forest species in northern Mesoamerica has been severely affected by Quaternary climatic fluctuations [59], [62]. Global climate model simulations and paleodata for Mesoamerica [59], [62], [68], [69] suggest that cloud forest species expanded into the lowlands during glacial cycles and that the distributions of these species contracted and fragmented during the interglacials. The observed temporal heterogeneity in divergence, spanning the Pliocene to the late Pleistocene, suggests that repeated cycles of cloud forest contraction and expansion due to Pleistocene climatic cycling, has shaped genetic divergence at these phylogeographic breaks. Given the single-locus nature of our data and that different strategies and choices of calibrations were used in our study, potential error in divergence time estimates may exist. Future studies that incorporate multi-locus data may be useful to produce more accurate and precise estimates of divergence [43], [70], with better model fits in msBayes that would likely lead to a stronger signal of asynchronous diversification.

### Cloud forest conservation

The cloud forests are the most threatened ecosystems at the regional level in Mesoamerica [1]. These forests are of great importance due to the extraordinary biodiversity they support and their importance in regional hydrology [71]. The highlands of northern Mesoamerica harbor among of the most endemic and vulnerable biodiversity on the planet, and they are one of Conservation International's recognized ‘Biodiversity Hotspots' [72]. According to the prioritized regions of cloud forest in Mexico identified by the Comisión Nacional para el Conocimiento y Uso de la Biodiversidad [71], we have identified distinct genetic lineages from Los Tuxtlas and Chiapas that require immediate and urgent conservation attention. These include populations of *L. styraciflua*, *C. curvipennis* ( = *C. excellens*), *B. brunneinucha*, *B. belli* and *C. ophthalmicus* from Los Tuxtlas that we found to be genetically distinct from populations distributed along the Sierra Madre Oriental. Furthermore, we found that Chiapan populations of *L. amethystinus* and *B. belli* separated by the Central Depression were also genetically distinct. Conservation of the Tuxtlas region is particularly important due to the restricted distribution of the cloud forest, and because of the accelerated deforestation rates in the Tuxtlas region [73] that threaten the endemic genetic diversity of these and possibly other co-distributed taxa. With the exception of the El Cielo Biosphere Reserve in Tamaulipas, most of our sampled populations along the Sierra Madre Oriental (San Luis Potosí, Hidalgo, Puebla, Veracruz, and Oaxaca) are unprotected, facing illegal logging impacts and impacts from conversion to pasture land for cattle, agricultural use and urban expansion [71]. Our study highlights the importance of these populations as reservoirs of endemic genetic diversity that merit conservation action.

## Conclusions

The results of this study, coupled with existing studies focusing on the region [14], [15], [19], [20], provides strong evidence that the evolutionary history of contemporary lineages inhabiting the cloud forests in northern Mesoamerica is complex and often lineage-specific. This complexity is likely due to differences among taxa in ecological niche requirements and dispersal capabilities. Our results provide the first broad taxonomic analysis of comparative phylogeographic data of cloud forest species in Mesoamerica, providing new insight into how this unique biota might be conserved. We find that while no single species is necessarily a good “marker” or “keystone” representative for the genetic diversity in other species, there is broad agreement among members of the cloud forest biome in delineating major areas of genetic endemism that are likely important for conservation and long-term persistence of populations with local adaptations.

## Supporting Information

Table S1 **Geographic location and GenBank accession numbers of the cloud forest species used in the study.**
(DOC)

Table S2 **Sample sizes, molecular markers, substitution models, substitution rates, and temporal calibrations used in this study.**
(DOC)

Table S3 **Summary statistics for sequence divergence by species.**
(DOC)

Text S1 **Details of nucleotide evolutionary models used, fossil and secondary calibrations, substitution rates and taxon sampling to divergence time estimation.**
(DOC)
