# Supplementary material for: Comparative Phylogeographic Analyses Illustrate the Complex Evolutionary History of Threatened Cloud Forests of Northern Mesoamerica
Source: PLoS One. 2013 Feb 7;8(2):e56283. doi: 10.1371/journal.pone.0056283 (PMC3567015; doi:10.1371/journal.pone.0056283)
Supplement: Table S1 — Geographic location and GenBank accession numbers of the cloud forest species used in the study. (DOC) [file pone.0056283.s001.doc]

**Table S1.** Geographic location and GenBank accession numbers of the cloud forest species used in the study. nSMO = northern Sierra Madre Oriental; cSMO = central Sierra Madre Oriental; sSMO = southern Sierra Madre Oriental; TUX = Los Tuxtlas region; cCHIS = central highlands of Chiapas; pCHIS = Pacific highlands of Chiapas; WEST = Guerrero, Michoacán, Jalisco, Tlaxcala, Estado de México, Morelos; MIA = Sierra de Miahuatlán, Oaxaca; YUC = Yucatan Peninsula; COL = Colombia; CR = Costa Rica; GUAT = Guatemala; SALV = El Salvador. Sequence sources (see full references in **Text S1**).

|  |  |  |  |  |  |  | |  |
| --- | --- | --- | --- | --- | --- | --- | --- | --- |
| **Population** | **Locality** | **Region** | ***n*** | **Altitude**  **(m a.s.l.)** | **Latitude N** | **Longitude W** | |  |
|  | *Podocarpus matudae*  GenBank acc. nos. HMO36081–HMO36102 [3], JX556873–JX556880, JX556900–JX556906 | | | | | |  | |
| 1 | México, Tamaulipas, El Cielo | nSMO | 9 | 1500 | 23° 03´ | 99° 13´ | |  |
| 2 | México, Hidalgo, Tlanchinol | nSMO | 11 | 2146 | 21° 01´ | 98° 38´ | |  |
| 3 | México, Hidalgo, Agua Blanca | nSMO | 10 | 1836 | 20° 22´ | 98° 21´ | |  |
| 4 | México, Puebla, Huauchinango | cSMO | 11 | 1500 | 20° 08´ | 98° 51´ | |  |
| 5 | México, Puebla, Lagunillas | cSMO | 9 | 1937 | 20° 13´ | 97° 57´ | |  |
| 6 | México, Veracruz, Huayacocotla | nSMO | 9 | 1840 | 20° 37´ | 98° 27´ | |  |
| 7 | México, Veracruz, Acatlán | cSMO | 20 | 1650 | 19° 40´ | 96° 51´ | |  |
| 8 | México, Veracruz, Santa Marta | TUX | 3 | 2302 | 18° 19´ | 94° 51´ | |  |
| 9 | México, Oaxaca, Teotitlán | sSMO | 11 | 2266 | 18° 10´ | 97° 00´ | |  |
| 10 | México, Oaxaca, Comaltepec | sSMO | 10 | 2478 | 17° 35´ | 96° 28´ | |  |
| 11 | México, Oaxaca, Zacatepec | sSMO | 11 | 1488 | 17° 09´ | 96° 01´ | |  |
| 12 | México, Chiapas, Montebello | cCHIS | 10 | 1900 | 16° 07´ | 91° 43´ | |  |
| 13 | México, Chiapas, Buenos Aires | cCHIS | 10 | 800 | 15° 19´ | 92° 16´ | |  |
| 14 | Guatemala, Puerto Barrios | GUAT | 12 | 1750 | 15° 39´ | 88° 40´ | |  |
| 15 | Guatemala, Verapaz, Purulha | GUAT | 9 | 953 | 15° 13´ | 90° 13´ | |  |
| 16 | México, Jalisco, El Tuito | WEST | 2 | 1300 | 20° 21´ | 105° 17´ | |  |
|  |  |  |  |  |  |  | |  |
|  | *Liquidambar styraciflua*  GenBank acc. nos. JX556867–JX556872 |  |  |  |  |  | |  |
| 1 | México, San Luis Potosí, Aquismón | nSMO | 2 | 378 | 21º 37´ 55´´ | 99º 03´ 34´´ | |  |
| 2 | México, San Luis Potosí, Xilitla | nSMO | 10 | 637 | 21º 22´ 39´´ | 98º 59´ 35´´ | |  |
| 3 | México, Hidalgo, San Bartolo Tutotepec | nSMO | 15 | 1155 | 20º 21´ 11´´ | 98º 13´ 10´´ | |  |
| 4 | México, Hidalgo, Tlanchinol | nSMO | 11 | 1476 | 20º 56´ 55´´ | 98º 04´ 12´´ | |  |
| 5 | México, Querétaro, Sierra Gorda, Neblinas | nSMO | 4 | 1130 | 21º 15´ 10´´ | 99º 04´ 31´´ | |  |
| 6 | México, Veracruz, Huayacocotla, Helechales | nSMO | 10 | 1950 | 20º 37´ 17´´ | 98º 27´ 49´´ | |  |
| 7 | México, Puebla, Huauchinango | cSMO | 14 | 1500 | 18º 12´ 49´´ | 98º 02´ 19´´ | |  |
| 8 | México, Puebla, Huitzilan | cSMO | 13 | 975 | 19º 57´ 29´´ | 97º 41´ 17´´ | |  |
| 9 | México, Veracruz, Coatepec, La Cortadura | cSMO | 10 | 2100 | 19º 29´ 05´´ | 97º 02´ 20´´ | |  |
| 10 | México, Veracruz, Huatusco | cSMO | 10 | 1215 | 19º 11´ 07´´ | 96º 57´ 32´´ | |  |
| 11 | México, Veracruz, Chiconquiaco | cSMO | 11 | 2005 | 19º 44´ 58´´ | 96º 48´ 54´´ | |  |
| 12 | México, Veracruz, San Andrés Tuxtla | TUX | 10 | 648 | 18º 28´ 46´´ | 95º 10´ 33´´ | |  |
| 13 | México, Oaxaca, Comaltepec | sSMO | 10 | 1198 | 17º 39´ 16´´ | 96º 20´ 13´´ | |  |
| 14 | México, Oaxaca, Sta. María Chayotepec | sSMO | 10 | 1137 | 16º 44´ 54´´ | 95º 27´ 24´´ | |  |
| 15 | México, Chiapas, Jitotol | cCHIS | 10 | 1692 | 17º 01´ 47´´ | 92º 50´ 46´´ | |  |
| 16 | México, Chiapas, La Trinitaria, L. Encantada | cCHIS | 10 | 1422 | 16º 07´ 41´´ | 91º 43´ 49´´ | |  |
| 17 | México, Chiapas, Angel Albino Corzo | pCHIS | 10 | 1471 | 15º 42´ 02´´ | 92º 43´ 48´´ | |  |
| 18 | México, Chiapas, Coapilla | pCHIS | 13 | 1793 | 17º 09´ 50´´ | 93º 08´ 50´´ | |  |
|  |  |  |  |  |  |  | |  |
|  | *Palicourea padifolia*  GenBank acc. nos. JF891318–JF891381 [14] |  |  |  |  |  | |  |
| 1 | México, Hidalgo, San Bartolo Tutotepec | nSMO | 5 | 1155 | 20º 21´ 11´´ | 98º 13´ 10´´ | |  |
| 2 | México, Hidalgo, Tlanchinol | nSMO | 3 | 1500 | 21º 01´11´´ | 98º 38´ 35´´ | |  |
| 3 | México, Hidalgo, Apantlazol | nSMO | 4 | 1422 | 20º 59´ 05´´ | 98º 37´ 38´´ | |  |
| 4 | México, Puebla, Apulco | cSMO | 7 | 405 | 19º 55´ 15´´ | 97º 06´ 24´´ | |  |
| 5 | México, Puebla, Cuetzalan | cSMO | 5 | 1319 | 19º 59´ 10´´ | 97º 30´ 35´´ | |  |
| 6 | México, Puebla, Huitzilan | cSMO | 8 | 1487 | 19º 57´ 29´´ | 97º 41´ 17´´ | |  |
| 7 | México, Veracruz, Aguita Fría | cSMO | 4 | 1500 | 19º 31´ 19´´ | 96º 59´ 21´´ | |  |
| 8 | México, Veracruz, La Martinica | cSMO | 7 | 1560 | 19º 35´ 17´´ | 96º 56´ 49´´ | |  |
| 9 | México, Veracruz, El Riscal | cSMO | 6 | 1586 | 19º 28´ 47´´ | 96º 59´ 51´´ | |  |
| 10 | México, Veracruz, La Pitaya | cSMO | 5 | 1346 | 19º 30´ 28´´ | 96º 57´ 36´´ | |  |
| 11 | México, Veracruz, Coapexpan | cSMO | 5 | 1392 | 19º 31´ 22´´ | 96º 58´ 02´´ | |  |
| 12 | México, Veracruz, Clavijero | cSMO | 5 | 1341 | 19º 30´ 47´´ | 96º 56´ 28´´ | |  |
| 13 | México, Veracruz, Xico | cSMO | 4 | 1350 | 19º 24´ 37´´ | 96º 59´ 37´´ | |  |
| 14 | México, Veracruz, Coacoatzintla | cSMO | 6 | 1040 | 19º 37´ 41´´ | 96º 52´ 56´´ | |  |
| 15 | México, Veracruz, Xolostla | cSMO | 6 | 1777 | 19º 31´ 36´´ | 97º 00´ 34´´ | |  |
| 16 | México, Veracruz, Huatusco | cSMO | 4 | 1418 | 19º 09´ 58´´ | 96º 58´ 07´´ | |  |
| 17 | México, Veracruz, Volcán de San Martín | TUX | 6 | 1111 | 18º 32´ 52´´ | 95º 11´ 21´´ | |  |
| 18 | México, Veracruz, Ejido Ruíz Cortinez | TUX | 5 | 998 | 18º 31´ 13´´ | 95º 09´ 34´´ | |  |
| 19 | México, Oaxaca, San Juan Yaguila | sSMO | 10 | 2112 | 17º 29´ 28´´ | 96º 22´ 50´´ | |  |
| 20 | México, Chiapas, Jitotol | cCHIS | 5 | 1692 | 17º 01´ 47´´ | 92º 50´ 46´´ | |  |
| 21 | México, Chiapas, Montebello | cCHIS | 8 | 1488 | 16º 07´ 07´´ | 91º 43´ 39´´ | |  |
| 22 | México, Chiapas, Buenos Aires | pCHIS | 4 | 1852 | 15º 19´ 42´´ | 92º 16´ 50´´ | |  |
|  |  |  |  |  |  |  | |  |
|  | *Moussonia deppeana*  GenBank acc. nos. JX847822–JX8447850, JX847851–JX847883 | | | | | |  | |
| 1 | México, San Luis Potosí, Aquismón | nSMO | 8 | 378 | 21º 37´ 55´´ | 99º 03´ 34´´ | |  |
| 2 | México, Hidalgo, Tlanchinol | nSMO | 9 | 1476 | 20º 56´ 55´´ | 98º 04´ 12´´ | |  |
| 3 | México, Hidalgo, Molango | nSMO | 9 | 1466 | 20º 56´ 28´´ | 98º 41´ 11´´ | |  |
| 4 | México, Hidalgo, Quetzalzongo | nSMO | 10 | 1155 | 20º 21´ 11´´ | 98º 13´ 10´´ | |  |
| 5 | México, Veracruz, Huayacocotla | nSMO | 10 | 1937 | 20º 37´ 18´´ | 98º 27´ 50´´ | |  |
| 6 | México, Puebla, Cuetzalan | cSMO | 11 | 906 | 20º 00´ 49´´ | 97º 30´ 21´´ | |  |
| 7 | México, Puebla, Huitzilan | cSMO | 9 | 975 | 19º 57´ 29´´ | 97º 41´ 17´´ | |  |
| 8 | México, Puebla, Apulco | cSMO | 7 | 1382 | 19º 55´ 06´´ | 97º 36´ 30´´ | |  |
| 9 | México, Veracruz, Clavijero | cSMO | 6 | 1225 | 19º 30´ 47´´ | 96º 56´ 28´´ | |  |
| 10 | México, Veracruz, Tlalnelhuayocan | cSMO | 7 | 1624 | 19º 34´ 47´´ | 96º 57´ 38´´ | |  |
| 11 | México, Veracruz, Araña Negra | cSMO | 1 | 1472 | 19º 31´ 37´´ | 96º 58´ 57´´ | |  |
| 12 | México, Veracruz, Coapexpan | cSMO | 10 | 1392 | 19º 31´ 36´´ | 96º 58´ 02´´ | |  |
| 13 | México, Veracruz, La Pitaya | cSMO | 7 | 1362 | 19º 30´ 28´´ | 96º 57´ 36´´ | |  |
| 14 | México, Veracruz, El Riscal | cSMO | 11 | 1586 | 19º 28´ 22´´ | 96º 59´ 51´´ | |  |
| 15 | México, Veracruz, camino a El Riscal | cSMO | 7 | 1452 | 19º 28´ 19´´ | 96º 59´ 51´´ | |  |
| 16 | México, Veracruz, Coatepec | cSMO | 5 | 1327 | 19º 28´ 43´´ | 96º 58´ 32´´ | |  |
| 17 | México, Veracruz, Xico | cSMO | 9 | 1350 | 19º 24´ 17´´ | 96º 59´ 31´´ | |  |
| 18 | México, Veracruz, Volcán San Martín | TUX | 9 | 1527 | 18º 33´ 44´´ | 95º 11´ 39´´ | |  |
| 19 | México, Veracruz, Ejido Ruíz Cortinez | TUX | 4 | 992 | 18º 32´ 03´´ | 95º 08´ 18´´ | |  |
| 20 | México, Oaxaca, Valle Nacional | sSMO | 5 | 603 | 17º 43´ 27´´ | 96º 19´ 36´´ | |  |
| 21 | México, Oaxaca, Valle Nacional2 | sSMO | 9 | 1198 | 17º 39´ 16´´ | 96º 20´ 13´´ | |  |
| 22 | México, Oaxaca, San Juan Yaguila | sSMO | 4 | 2300 | 17º 29´ 21´´ | 96º 22´ 38´´ | |  |
| 23 | México, Oaxaca, Santiago | sSMO | 3 | 1118 | 16º 44´ 34´´ | 95º 27´ 24´´ | |  |
| 24 | México, Oaxaca, Cerro Baúl | sSMO | 3 | 1339 | 16º 32´ 41´´ | 94º 10´ 40´´ | |  |
| 25 | México, Oaxaca, Cerro Piedra Larga | MIA | 4 | 1257 | 16º 09´ 35´´ | 96º 59´ 52´´ | |  |
| 26 | México, Oaxaca, El Polvorín | MIA | 2 | 1647 | 16º 11´ 46´´ | 97º 07´ 27´´ | |  |
| 27 | México, Oaxaca, San Rafael | MIA | 3 | 958 | 16º 09´ 12´´ | 97º 04´ 10´´ | |  |
| 28 | México, Chiapas, Jitotol | cCHIS | 8 | 1692 | 17º 01´ 47´´ | 92º 50´ 46´´ | |  |
| 29 | México, Chiapas, San Cristobal de las Casas | cCHIS | 11 | 1864 | 16º 43´ 11´´ | 92º 49´ 19´´ | |  |
| 30 | México, Chiapas, Nueva Colombia | pCHIS | 8 | 1607 | 15º 43´ 03´´ | 92º 44´ 14´´ | |  |
| 31 | México, Chiapas, Vega del Rosario | pCHIS | 9 | 1772 | 15º 30´ 42´´ | 92º 17´ 46´´ | |  |
| 32 | México, Chiapas, Volcán Tacaná | pCHIS | 4 | 1753 | 15º 05´ 34´´ | 92º 05´ 31´´ | |  |
| 33 | México, Chiapas, Volcán Tacaná2 | pCHIS | 6 | 2392 | 15º 06´ 13´´ | 92º 06´ 07´´ | |  |
| 34 | Guatemala, Palopó | GUAT | 6 | 1800 | 14º 37´ 19´´ | 91º 08´ 04´´ | |  |
| 35 | Jalisco, Sierra de Manantlán, Las Joyas | WEST | 4 | 1800 | 19º 31´ 42´´ | 104º 10´ 46´´ | |  |
| 36 | Jalisco, Sierra de Manantlán, San Campús | WEST | 5 | 1990 | 19º 31´ 42´´ | 104º 10´ 46´´ | |  |
|  |  |  |  |  |  |  | |  |
|  | *Rhipsalis baccifera*  GenBank acc. nos. JX556881–JX556899 |  |  |  |  |  | |  |
| 1 | México, Tamaulipas, Gómez Farías | nSMO | 7 | 564 | 23º 02´ 40´´ | 99º 10´ 09´´ | |  |
| 2 | México, San Luis Potosí, Aquismón1 | nSMO | 10 | 691 | 21º 35´ 30´´ | 99º 04´ 59´´ | |  |
| 3 | México, San Luis Potosí, Aquismón2 | nSMO | 9 | 378 | 21º 37´ 55´´ | 99º 03´ 34´´ | |  |
| 4 | México, San Luis Potosí, Tamazunchale | nSMO | 5 | 172 | 21º 15´ 04´´ | 98º 45´ 46´´ | |  |
| 5 | México, Hidalgo, Huejutla | nSMO | 9 | 348 | 21º 03´ 31´´ | 98º 30´ 39´´ | |  |
| 6 | México, Veracruz, Uxpanapa, Nva. Córdoba | nSMO | 3 | 662 | 17º 14´ 27´´ | 94º 45´ 19´´ | |  |
| 7 | México, Puebla, Cuetzalan | cSMO | 8 | 906 | 20º 00´ 49´´ | 97º 30´ 21´´ | |  |
| 8 | México, Puebla, Huauchinango | cSMO | 10 | 1500 | 18º 12´ 49´´ | 98º 02´ 19´´ | |  |
| 9 | México, Veracruz, Clavijero | cSMO | 9 | 1225 | 20º 30´ 47´´ | 97º 56´ 28´´ | |  |
| 10 | México, Veracruz, Tlalnelhuayocan | cSMO | 4 | 1624 | 19º 34´ 47´´ | 96º 57´ 38´´ | |  |
| 11 | México, Veracruz, La Pitaya | cSMO | 12 | 1362 | 19º 30´ 28´´ | 96º 57´ 36´´ | |  |
| 12 | México, Veracruz, El Riscal | cSMO | 9 | 1586 | 19º 28´ 22´´ | 96º 59´ 51´´ | |  |
| 13 | México, Veracruz, Xico | cSMO | 13 | 1350 | 19º 24´ 17´´ | 96º 59´ 31´´ | |  |
| 14 | México, Veracruz, Choapas, El Roble | sSMO | 2 | 18 | 17º 38´ 41´´ | 93º 58´ 54´´ | |  |
| 15 | México, Veracruz, Choapas, R. La Lima | sSMO | 2 | 31 | 17º 23´ 29´´ | 93º 44´ 00´´ | |  |
| 16 | México, Veracruz, Choapas, El Jobo | sSMO | 2 | 153 | 17º 39´ 54´´ | 94º 00´ 42´´ | |  |
| 17 | México, Veracruz, Volcán San Martín | TUX | 8 | 1527 | 18º 28´ 46´´ | 95º 10´ 33´´ | |  |
| 18 | México, Veracruz, Playa Escondida | TUX | 2 | 20 | 18 º 36´ 15´´ | 95º 05´ 33´´ | |  |
| 19 | México, Oaxaca, Tres Marías | sSMO | 14 | 74 | 17º 47´ 28´´ | 96º 16´ 02´´ | |  |
| 20 | México, Oaxaca, Sta. María Chayotepec | sSMO | 3 | 1137 | 16º 44´ 54´´ | 95º 27´ 24´´ | |  |
| 21 | México, Chiapas, Ocozocuautla | cCHIS | 8 | 665 | 16º 57´ 01´´ | 93º 27´ 08´´ | |  |
| 22 | Guatemala, Patutul, Finca Agrícola | GUAT | 5 | 260 | 14º 22´ 24´´ | 91º 08´ 18´´ | |  |
| 23 | Guatemala, Patutul, Santa Fé | GUAT | 7 | 395 | 14º 27´ 07´´ | 91º 08´ 30´´ | |  |
| 24 | Colombia, Quindio | COL | 1 | 1237 | 4º 31´ 18´´ | 75º 48´ 06´´ | |  |
|  |  |  |  |  |  |  | |  |
|  | *Campylopterus curvipennis*  GenBank acc. nos. HQ380686–HQ380755 [26] |  |  |  |  |  | |  |
| 1 | México, Tamaulipas, El Cielo | nSMO | 17 | 943 | 25º 03´ 33´´ | 99º 12´ 21´´ | |  |
| 2 | México, Tamaulipas, Gomez Farías | nSMO | 4 | 564 | 23º 03´ 58´´ | 99º 10´ 06´´ | |  |
| 3 | México, San Luis Potosí, El Naranjo | nSMO | 6 | 270 | 22º 34´ 33´´ | 99º 21´ 11´´ | |  |
| 4 | México, San Luis Potosí, Aquismón | cSMO | 4 | 378 | 21º 37´ 30´´ | 99º 01´ 12´´ | |  |
| 5 | México, San Luis Potosí, Xilitla | cSMO | 8 | 637 | 21º 22´ 39´´ | 98º 59´ 35´´ | |  |
| 6 | México, Hidalgo, San Bartolo Tutotepec | cSMO | 1 | 1155 | 20º 21´ 11´´ | 98º 13´ 10´´ | |  |
| 7 | México, Puebla, Cuetzalan | sSMO | 27 | 906 | 20º 00´ 49´´ | 97º 30´ 21´´ | |  |
| 8 | México, Veracruz, Macuiltépetl | sSMO | 3 | 1500 | 19º 32´ 50´´ | 96º 55´ 12´´ | |  |
| 9 | México, Veracruz, Coapexpan | sSMO | 6 | 1392 | 19º 31´ 22´´ | 96º 58´ 02´´ | |  |
| 10 | México, Veracruz, Parque Clavijero | sSMO | 7 | 1225 | 19º 30´ 47´´ | 96º 56´ 28´´ | |  |
| 11 | México, Veracruz, La Orduña | sSMO | 21 | 1190 | 19º 27´ 50´´ | 96º 56´ 13´´ | |  |
| 12 | México, Veracruz, El Riscal | sSMO | 2 | 1586 | 19º 28´ 47´´ | 96º 59´ 51´´ | |  |
| 13 | México, Veracruz, Ursulo Galván | sSMO | 13 | 1200 | 19º 25´ 31´´ | 96º 58´ 35´´ | |  |
| 14 | México, Veracruz, Xico | sSMO | 4 | 1350 | 19º 24´ 37´´ | 96º 59´ 31´´ | |  |
| 15 | México, Veracruz, Amatlán | sSMO | 3 | 720 | 18º 49´ 51´´ | 96º 54´ 07´´ | |  |
| 16 | México, Veracruz, Los Tuxtlas | TUX | 10 | 998 | 18º 33´ 29´´ | 95º 11´ 46´´ | |  |
| 17 | México, Veracruz, El Nopal | TUX | 1 | 664 | 17º 14´ 23´´ | 90º 45´ 40´´ | |  |
| 18 | México, Veracruz, Chalchijapa | TUX | 1 | 260 | 17º 02´ 04´´ | 94º 41´ 57´´ | |  |
| 19 | México, Campeche, Escárcega | YUC | 1 | 60 | 18º 38´ 12´´ | 90º 47´ 16´´ | |  |
| 20 | México, Campeche, Río Bec | YUC | 2 | 251 | 18º 24´ 31´´ | 89º 26´ 37´´ | |  |
| 21 | México, Campeche, Ejido 20 de Noviembre | YUC | 15 | 179 | 18º 25´ 29´´ | 89º 18´ 37´´ | |  |
| 22 | México, Quintana Roo, Tres Garantías | YUC | 3 | 137 | 18º 12´ 51´´ | 89º 02´ 34´´ | |  |
|  |  |  |  |  |  |  | |  |
|  | *Amazilia cyanocephala*  GenBank acc. nos. JX050059–JX050109[28] |  |  |  |  |  | |  |
| 1 | México, Tamaulipas, Alta Cima | nSMO | 4 | 943 | 23º 03´ | 99º 12´ | |  |
| 2 | México, Hidalgo, Picuatla | cSMO | 2 | 1155 | 20º 56´ | 98º 32´ | |  |
| 3 | México, Hidalgo, Tolantongo | cSMO | 0 | 1476 | 20º 38´ | 98º 58´ | |  |
| 4 | México, Hidalgo, Oxpantla | cSMO | 1 | 1697 | 20º 40´ | 98º 38 | |  |
| 5 | México, Puebla, Lagunillas | cSMO | 15 | 1500 | 20º 13´ | 97º 57´ | |  |
| 6 | México, Puebla, Cuetzalan | cSMO | 7 | 1319 | 19º 59´ | 97º 30´ | |  |
| 7 | México, Puebla, Huitzilan | cSMO | 6 | 1487 | 19º 57´ | 97º 41´ | |  |
| 8 | México, Veracruz, Riscal | cSMO | 6 | 1586 | 19º 28´ | 96º 59´ | |  |
| 9 | México, Veracruz, La Orduña | cSMO | 3 | 1349 | 19º 27´ | 96º 56´ | |  |
| 10 | México, Veracruz, Coapexpan | cSMO | 6 | 1392 | 19º 31´ | 96º 58´ | |  |
| 11 | México, Veracruz, Clavijero | cSMO | 11 | 1341 | 19º 30´ | 96º 56´ | |  |
| 12 | México, Veracruz, La Pitaya | cSMO | 2 | 1346 | 19º 30´ | 96º 57´ | |  |
| 13 | México, Veracruz, Macuiltépetl | cSMO | 4 | 1481 | 19º 32´ | 96º 55´ | |  |
| 14 | México, Veracruz, Coatepec | cSMO | 7 | 1190 | 19º 27´ | 96º 57´ | |  |
| 15 | México, Veracruz, Huatusco | cSMO | 2 | 1418 | 19º 11´ | 96º 58´ | |  |
| 16 | México, Oaxaca, San Martín Caballero | sSMO | 4 | 1930 | 18º 06´ | 96º 38´ | |  |
| 17 | México, Oaxaca, Cerro Baúl | pCHIS | 6 | 1137 | 16º 30´ | 94º 11´ | |  |
| 18 | México, Chiapas, Pueblo Nuevo | cCHIS | 10 | 1639 | 17º 08´ | 92º 53´ | |  |
| 19 | México, Chiapas, Jitotol | cCHIS | 10 | 1642 | 17º 08´ | 92º 52´ | |  |
| 20 | México, Chiapas, Montebello | cCHIS | 11 | 1422 | 16º 07´ | 91º 43´ | |  |
| 21 | México, Chiapas, Salvador Urbina | pCHIS | 13 | 908 | 15º 45´ | 92º 49´ | |  |
|  |  |  |  |  |  |  | |  |
|  | *Lampornis amethystinus*  GenBank acc. nos. EU543284–EU543433 [27], JX847800–JX847807, JX847808–JX847821 | | | | | |  | |
| 1 | México, Tamaulipas, Alta Cima | nSMO | 2 | 943 | 25º 03´ 33´´ | 99º 12´ 21´´ | |  |
| 2 | México, Querétaro, El Pemoche^27^ | nSMO | 2 |  | 21º 13´ 34´´ | 99º 06´ 34´´ | |  |
| 3 | México, Hidalgo, Tlanchinol a México^27^ | nSMO | 6 | 1129 | 21º 01´ 24´´ | 98º 36´ 37´´ | |  |
| 4 | México, Hidalgo, Tlanchinol a Pachuca | nSMO | 2 | 1476 | 20º 56´ 55´´ | 98º 04´ 12´´ | |  |
| 5 | México, Hidalgo, Tenango de Doria^27^ | nSMO | 5 |  | 20º 19´ 10´´ | 98º 13´ 18´´ | |  |
| 6 | México, Puebla, La Galera | cSMO | 5 | 950 | 19º 59´ 10´´ | 97º 36´ 35´´ | |  |
| 7 | México, Puebla, Huitzilan | cSMO | 1 | 975 | 19º 57´ 29´´ | 91º 41´ 17´´ | |  |
| 8 | México, Puebla, Lagunillas | cSMO | 2 | 1500 | 18º 12´ 49´´ | 98º 02´ 19´´ | |  |
| 9 | México, Veracruz, Zacualpan^27^ | cSMO | 5 |  | 20º 28´ 01´´ | 98º 18´ 52´´ | |  |
| 10 | México, Veracruz, El Riscal | cSMO | 5 | 1586 | 19º 28´ 22´´ | 96º 59´ 51´´ | |  |
| 11 | México, Veracruz, Clavijero | cSMO | 1 | 1341 | 19º 30´ | 96º 56´ | |  |
| 12 | México, Veracruz, Santa Marta^27^ | TUX | 4 | 1613 | 18º 20´ 45´´ | 94º 51´ 28´´ | |  |
| 13 | México, Oaxaca, Comaltepec | sSMO | 2 |  | 17º 26´ 42´´ | 96º 29´ 47´´ | |  |
| 14 | México, Oaxaca, Puerto de la Soledad^27^ | sSMO | 3 |  | 17º 02´ 15´´ | 96º 59´ 45´´ | |  |
| 15 | México, Oaxaca, Peña Verde^27^ | sSMO | 2 |  | 17º 50´ 42´´ | 96º 44´ 24´´ | |  |
| 16 | México, Oaxaca, San Martín Caballero^27^ | sSMO | 4 |  | 18º 06´ 41´´ | 96º 38´ 24´´ | |  |
| 17 | México, Oaxaca, Cerro de Zempoaltepec^27^ | sSMO | 2 |  | 17º 08´ 00´´ | 96º 01´ 00´´ | |  |
| 18 | México, Oaxaca, Cerro Piedra Larga^27^ | MIA | 3 | 1349 | 16º 09´ 40´´ | 97º 00´ 37´´ | |  |
| 19 | México, Chiapas, Pueblo Nuevo^27^ | cCHIS | 3 |  | 17º 10´ 59´´ | 92º 04´ 59´´ | |  |
| 20 | México, Chiapas, Jitotol | cCHIS | 4 | 1692 | 17º 01´ 47´´ | 92º 50´ 46´´ | |  |
| 21 | México, Chiapas, San Cristobal de las Casas^27^ | cCHIS | 2 |  | 16º 49´ 00´´ | 92º 35´ 00´´ | |  |
| 22 | México, Chiapas, Huitepec^27^ | cCHIS | 3 |  | 16º 47´ 30´´ | 92º 45´ 24´´ | |  |
| 23 | México, Chiapas, El Triunfo^27^ | pCHIS | 1 |  | 15º 37´ 00´´ | 92º 50´ 00´´ | |  |
| 24 | México, Chiapas, Volcán Tacaná^27^ | pCHIS | 2 |  | 15º 04´ 00´´ | 92º 05´ 00´´ | |  |
| 25 | Guatemala, S. de las Minas, Quetzaltenango^27^ | GUAT | 2 |  | 14º 46´ 40´´ | 91º 40´ 53´´ | |  |
| 26 | El Salvador, Chalatenango^27^ | GUAT | 1 |  | 14º 01´ 51´´ | 88º 52´ 33´´ | |  |
| 27 | México, Jalisco, Sierra de Manantlán | WEST | 4 | 1990 | 19º 31´ 42´´ | 104º 10´ 46´´ | |  |
| 28 | México, Jalisco, Nevado de Colima | WEST | 1 |  | 19º 31´ 42´´ | 103º 37´ 00´´ | |  |
| 29 | México, Michoacán, Zirimondiro^27^ | WEST | 2 |  | 19º 36´ 00´´ | 102º 20´ 17´´ | |  |
| 30 | México, Tlaxcala, La Malinche | WEST | 1 | 2900 | 19º 14´ 41´´ | 98º 06´ 58´´ | |  |
| 31 | México, Edo. de México, Ocuilan de Arteaga^27^ | WEST | 4 |  | 18º 56´ 42´´ | 99º 15´ 53´´ | |  |
| 32 | México, Michoacán, Coalcomán^27^ | WEST | 3 |  | 18º 48´ 24´´ | 102º 57´ 05´´ | |  |
| 33 | México, Guerrero, Carrizal de Bravo^27^ | WEST | 4 |  | 17º 36´ 00´´ | 99º 50´ 00´´ | |  |
| 34 | México, Guerrero, El Iris^27^ | WEST | 6 |  | 17º 29´ 00´´ | 100º 12´ 00´´ | |  |
|  |  |  |  |  |  |  | |  |
|  | *Lepidocolaptes affinis*  GenBank acc. nos. HQ014479–HQ014562 [31] |  |  |  |  |  | |  |
| 1 | México, San Luis Potosí, Xilitla | nSMO | 3 |  | 21.40 | 99.04 | |  |
| 2 | México, Querétaro, Laguna de la Cruz | nSMO | 1 |  | 21.36 | 99.51 | |  |
| 3 | México, Querétaro, Tres Lagunas | nSMO | 1 |  | 31.33 | 99.11 | |  |
| 4 | México, Querétaro, El Pemoche | nSMO | 1 |  | 21.23 | 99.11 | |  |
| 5 | México, Querétaro, Santa Inés | nSMO | 1 |  | 21.18 | 99.13 | |  |
| 6 | México, Querétaro, Jalpan | nSMO | 1 |  | 21.17 | 99.04 | |  |
| 7 | México, Hidalgo, Chilijapa | nSMO | 2 |  | 21.01 | 98.87 | |  |
| 8 | México, Hidalgo, Tlanchinol | nSMO | 3 |  | 20.99 | 98.07 | |  |
| 9 | México, Hidalgo, Cerro Jarros | nSMO | 2 |  | 20.99 | 99.14 | |  |
| 10 | México, Hidalgo, El Coyol | nSMO | 2 |  | 21.06 | 99.99 | |  |
| 11 | México, Hidalgo, Tenango de Doria | nSMO | 5 |  | 20.34 | 99.23 | |  |
| 12 | México, Puebla, Xocoyolo | cSMO | 1 |  | 19.98 | 97.55 | |  |
| 13 | México, Puebla, Zacapoaxtla | cSMO | 1 |  | 19.83 | 97.57 | |  |
| 14 | México, Veracruz, Sierra de Santa Marta | TUX | 1 |  | 18.32 | 94.83 | |  |
| 15 | México, Oaxaca, Puerto de la Soledad | sSMO | 2 |  | 18.17 | 97.00 | |  |
| 16 | México, Oaxaca, San Martín Caballero | sSMO | 1 |  | 18.11 | 96.64 | |  |
| 17 | México, Oaxaca, San Juan Yaguila | sSMO | 6 |  | 17.49 | 96.37 | |  |
| 18 | México, Oaxaca, San Juan Bautista | sSMO | 2 |  | 17.54 | 96.74 | |  |
| 19 | México, Oaxaca, Reyes Llano Grande | sSMO | 1 |  | 17.03 | 97.80 | |  |
| 19 | México, Oaxaca, Cerro Piedra Larga | MIA | 7 |  | 16.61 | 95.80 | |  |
| 20 | México, Guerrero, Atoyac | WEST | 1 |  | 17.48 | 100.20 | |  |
| 21 | México, Guerrero, Carrizal de Bravo | WEST | 1 |  | 17.82 | 99.97 | |  |
| 22 | México, Guerrero, Nueva Delhi | WEST | 1 |  | 17.42 | 100.20 | |  |
| 23 | México, Guerrero, Los Otates | WEST | 1 |  | 17.60 | 99.85 | |  |
| 24 | México, Chiapas, Coapilla | pCHIS | 6 |  | 17.17 | 93.15 | |  |
| 25 | México, Chiapas, Volcán Tacaná | pCHIS | 1 |  | 15.01 | 92.10 | |  |
| 26 | Guatemala, Quetzaltenango, Sta. María | GUAT | 2 |  | 14.72 | 91.53 | |  |
| 27 | El Salvador, Chalatenango, La Laguna | SALV | 4 |  | 14.13 | 88.92 | |  |
| 28 | El Salvador, Chalatenango, Metapán | SALV | 4 |  | 14.41 | 89.36 | |  |
| 29 | El Salvador, V. San Vicente, Nvo. Tepatitán | SALV | 7 |  | 13.60 | 88.84 | |  |
|  |  |  |  |  |  |  | |  |
|  | *Buarremon brunneinucha*  GenBank acc. nos. EU364902–EU364975 [30] |  |  |  |  |  | |  |
| 1 | México, Hidalgo, Tlanchinol | nSMO | 4 |  |  |  | |  |
| 2 | México, Hidalgo, Tenango de Doria | nSMO | 2 |  |  |  | |  |
| 3 | México, Hidalgo, Cerro Jarros | nSMO | 2 |  |  |  | |  |
| 4 | México, Hidalgo, El Coyol | nSMO | 1 |  |  |  | |  |
| 5 | México, Puebla, Cuetzalan | cSMO | 1 |  |  |  | |  |
| 6 | México, Puebla, Teziutlán | cSMO | 1 |  |  |  | |  |
| 7 | México, Puebla, Zacapoaxtla | cSMO | 2 |  |  |  | |  |
| 8 | México, Veracruz, Teocelo | cSMO | 2 |  |  |  | |  |
| 9 | México, Veracruz, Volcán Santa Marta | TUX | 2 |  |  |  | |  |
| 10 | México, Oaxaca, Los Chimalapas | sSMO | 1 |  |  |  | |  |
| 11 | México, Oaxaca, Cerro Peña Verde | sSMO | 1 |  |  |  | |  |
| 12 | México, Oaxaca, La Clemencia | sSMO | 1 |  |  |  | |  |
| 13 | México, Oaxaca, Puerto de la Soledad | sSMO | 3 |  |  |  | |  |
| 14 | México, Oaxaca, Cerro Zempoaltepetl | sSMO | 3 |  |  |  | |  |
| 15 | México, Oaxaca, Cerro Piedra Larga | MIA | 2 |  |  |  | |  |
| 16 | México, Oaxaca, Reyes Llano Grande | sSMO | 3 |  |  |  | |  |
| 17 | México, Guerrero, Carrizal de Bravo | WEST | 3 |  |  |  | |  |
| 18 | México, Guerrero, El Iris | WEST | 3 |  |  |  | |  |
| 19 | México, Michoacán, La Verdura | WEST | 1 |  |  |  | |  |
| 20 | México, Edo. México, Ocuilan | WEST | 1 |  |  |  | |  |
| 21 | México, Chiapas, Volcán Tacaná | pCHIS | 2 |  |  |  | |  |
| 22 | Guatemala, Quetzaltenango, El Baúl | GUAT | 1 |  |  |  | |  |
| 23 | Guatemala, Quetzaltenango, F. Sta. María | GUAT | 1 |  |  |  | |  |
|  |  |  |  |  |  |  | |  |
|  | *Basileuterus belli*  GenBank acc. nos. JX626333–JX626402 |  |  |  |  |  | |  |
| 1 | México, Hidalgo, 7 km E de Tlanchinol | nSMO | 4 | 1528 | 20º 59´ 15´´ | 98º 39´ 38´´ | |  |
| 2 | México, Hidalgo, Tenango de Doria | nSMO | 5 | 1656 | 20º 20´ 15´´ | 98º 13´ 32´´ | |  |
| 3 | México, Hidalgo, 4 km E de Eloxochitlan | nSMO | 3 | 1963 | 20º 44´ 45´´ | 98º 48´ 34´´ | |  |
| 4 | México, Querétaro, El Chuvaje | nSMO | 1 | 1519 | 21º 10´ 06´´ | 99º 33´ 06´´ | |  |
| 5 | México, Querétaro, 7 Km S de Tres Lagunas | nSMO | 2 | 1346 | 21º 11´ 23´´ | 99º 05´ 44´´ | |  |
| 6 | México, Veracruz, Zacualpan 6 Km E | cSMO | 1 | 1682 | 20º 25´ 54´´ | 98º 20´ 56´´ | |  |
| 7 | México, Veracruz, Los Tuxtlas | TUX | 5 | 1354 | 18º 32´ 59´´ | 95º 11´ 58´´ | |  |
| 8 | México, Oaxaca, Cerro Piedra Larga | MIA | 10 | 1757 | 16º 36´ 42´´ | 95º 45´ 20´´ | |  |
| 9 | México, Oaxaca, Sierra de Huautla | sSMO | 5 | 1890 | 18º 09´ 56´´ | 96º 49´ 59´´ | |  |
| 10 | México, Chiapas, Volcan Tacaná, Rio Mala | cCHIS | 5 | 1320 | 15º 03´ 45´´ | 92º 04´ 50´´ | |  |
| 11 | México, Chiapas, carr. Copainola-Ocutepec | cCHIS | 11 | 1843 | 17º 10´08´´ | 93º 08´ 43´´ | |  |
| 12 | México, Chiapas, El Triunfo | pCHIS | 5 | 1991 | 15º 39´ 29´´ | 92º 48´ 32´´ | |  |
| 13 | México, Edo. México, Ocuilan-Cuernavaca | WEST | 7 | 2348 | 18º 58´ 45´´ | 99º 24´ 59´´ | |  |
| 14 | México, Guerrero, Carrizal de Bravo | WEST | 5 | 2394 | 17º 37´ 17´´ | 99º 50´ 17´´ | |  |
| 15 | México, Jalisco, San Sebastián del Oeste | WEST | 3 | 1791 | 20º 44´ 57´´ | 104º 49´ 24´´ | |  |
|  |  |  |  |  |  |  | |  |
|  | *Chlorospingus ophthalmicus*  GenBank acc. nos. EU594945–EU595009 [29], JQ230883–JQ230963 | | | | | | | |
| 1 | México, Hidalgo, Tlanchinol^29^ | nSMO | 13 | 1413 | 21º 01´ 11´´ | 98º 38´ 35´´ | |  |
| 2 | México, Hidalgo, El Potrero^29^ | nSMO | 8 | 1967 | 20º 19´ 01´´ | 98º 13´ 17´´ | |  |
| 3 | México, Hidalgo, La Mojonera^29^ | nSMO | 3 | 1880 | 20º 38´ 17´´ | 98º 34´ 30´´ | |  |
| 4 | México, Hidalgo, Cerro Jarros | nSMO | 2 | 1836 | 21º 00´ 00´´ | 99º 07´ 59´´ | |  |
| 5 | México, Hidalgo, El Coyol | nSMO | 1 | 664 | 21º 04´ 05´´ | 98º 59´ 35´´ | |  |
| 6 | México, Querétaro, Tres Lagunas^29^ | nSMO | 1 | 1955 | 21º 16´ 35´´ | 99º 07´ 30´´ | |  |
| 7 | México, Querétaro, Santa Inés^29^ | nSMO | 2 | 1294 | 21º 10´ 39´´ | 99º 07´ 34´´ | |  |
| 8 | México, Querétaro, El Pemoche | nSMO | 1 | 1365 | 21º 13´ 34´´ | 99º 06´ 34´´ | |  |
| 9 | México, Puebla, Cuetzalan | cSMO | 5 | 799 | 20º 02´ 17´´ | 97º 31´ 35´´ | |  |
| 10 | México, Puebla, San Andrés Tlahualcingo | cSMO | 1 | 1349 | 20º 00´ 36´´ | 97º 50´ 17´´ | |  |
| 11 | México, Puebla, Jonotla^29^ | cSMO | 2 | 914 | 20º 01´ 48´´ | 97º 37´ 23´´ | |  |
| 12 | México, Puebla, Lagunillas | cSMO | 4 | 1051 | 18º 29´ 44´´ | 98º 40´ 13´´ | |  |
| 13 | México, Puebla, La Galera | cSMO | 5 | 950 | 19º 59´ 10´´ | 97º 36´ 35´´ | |  |
| 14 | México, Veracruz, Zacualpan | cSMO | 7 | 1572 | 20º 28´ 01´´ | 98º 18´ 52´´ | |  |
| 15 | México, Veracruz, El Riscal | cSMO | 9 | 1557 | 19º 28´ 47´´ | 96º 59´ 51´´ | |  |
| 16 | México, Veracruz, Coapexpan | cSMO | 9 | 1457 | 19º 31´ 22´´ | 96º 56´ 28´´ | |  |
| 17 | México, Veracruz, Clavijero | cSMO | 9 | 1341 | 19º 30´ 47´´ | 96º 56´ 28´´ | |  |
| 18 | México, Veracruz, Xico | cSMO | 9 | 1257 | 19º 24´ 37´´ | 96º 59´ 37´´ | |  |
| 19 | México, Veracruz, La Orduña | cSMO | 10 | 1199 | 19º 27´ 50´´ | 96º 56´ 13´´ | |  |
| 20 | México, Veracruz, Ejido Ruíz Cortinez | TUX | 15 | 1102 | 18º 31´ 13´´ | 95º 09´ 34´´ | |  |
| 21 | México, Oaxaca, San Juan Yaguila | sSMO | 11 | 2112 | 17º 29´ 12´´ | 96º 22´ 23´´ | |  |
| 22 | México, Oaxaca, San Martín Caballero | sSMO | 1 | 1423 | 18º 06´ 41´´ | 96º 38´ 24´´ | |  |
| 23 | México, Oaxaca, Puente Fierro^29^ | sSMO | 1 | 1370 | 18º 10´ 12´´ | 96º 50´ 47´´ | |  |
| 24 | México, Oaxaca, Peña Verde | sSMO | 5 | 1614 | 17º 50´ 42´´ | 96º 44´ 24´´ | |  |
| 25 | México, Oaxaca, Ejido Clemencia | sSMO | 3 | 357 | 18º 15´ 24´´ | 96º 44´ 18´´ | |  |
| 26 | México, Oaxaca, Puerto Soledad^29^ | sSMO | 18 | 2384 | 17º 02´ 15´´ | 96º 59´ 45´´ | |  |
| 27 | México, Oaxaca, La Esperanza | sSMO | 7 | 2902 | 17º 39´ 16´´ | 96º 20´ 13´´ | |  |
| 28 | México, Oaxaca, Reyes Llano Grande | sSMO | 1 | 1675 | 17º 01´ 30´´ | 97º 47´ 42´´ | |  |
| 29 | México, Guerrero, Carrizal de Bravo | WEST | 5 | 1126 | 17º 16´ 01´´ | 99º 43´ 59´´ | |  |
| 30 | México, Chiapas, Jitotol | cCHIS | 4 | 1575 | 17º 03´ 10´´ | 92º 51´ 15´´ | |  |
| 31 | México, Chiapas, Pueblo Nuevo | cCHIS | 7 | 482 | 17º 10´ 59´´ | 92º 04´ 59´´ | |  |
| 32 | México, Chiapas, Montebello | pCHIS | 14 | 569 | 16º 02´ 48´´ | 92º 29´ 49´´ | |  |
| 33 | México, Chiapas, Volcán Tacaná | pCHIS | 6 | 3992 | 15º 07´ 53´´ | 92º 06´ 36´´ | |  |
| 34 | México, Chiapas, El Triunfo | pCHIS | 4 | 1642 | 15º 43´ 03´´ | 92º 44´ 14´´ | |  |
| 35 | México, Chiapas, Nueva Colombia | pCHIS | 4 | 1755 | 15º 42´ 37´´ | 92º 44´ 15´´ | |  |
|  |  |  |  |  |  |  | |  |
|  | *Habromys* *lophurus* complex  GenBank acc. nos. DQ793090–DQ793118 [33] |  |  |  |  |  | |  |
|  | *Habromys simulatus* |  |  |  |  |  | |  |
| 1 | México, Hidalgo, Tenango de Doria | nSMO | 4 |  |  |  | |  |
| 2 | México, Veracruz, Zacualpan | cSMO | 1 |  |  |  | |  |
| 3 | México, Oaxaca, Cuicatlán, Pto. Soledad | sSMO | 4 |  |  |  | |  |
|  | *Habromys delicatus* |  |  |  |  |  | |  |
| 4 | México, Estado de México, Jilotepec | cSMO | 2 |  |  |  | |  |
|  | *Habromys schmidlyi* |  |  |  |  |  | |  |
| 5 | México, Estado de México, Zacualpan | cSMO | 2 |  |  |  | |  |
|  | *Habromys chinanteco* |  |  |  |  |  | |  |
| 6 | México, Oaxaca, Vista Hermosa | sSMO | 2 |  |  |  | |  |
|  | *Habromys lepturus* |  |  |  |  |  | |  |
| 7 | México, Oaxaca, Cerro Zempoaltepetl | sSMO | 4 |  |  |  | |  |
|  | *Habromys ixtlani* |  |  |  |  |  | |  |
| 8 | México, Oaxaca, Llano de las Flores | sSMO | 1 |  |  |  | |  |
| 9 | México, Oaxaca, Ixtlán | sSMO | 1 |  |  |  | |  |
|  | *Habromys lophurus* |  |  |  |  |  | |  |
| 10 | México, Chiapas, Volcán Tacaná | pCHIS | 2 |  |  |  | |  |
| 11 | México, Chiapas, El Triunfo | pCHIS | 1 |  |  |  | |  |
| 12 | Guatemala, Sierra de las Minas, San Lorenzo | GUAT | 1 |  |  |  | |  |
| 13 | Guatemala, Sierra de las Minas, La Cabaña | GUAT | 2 |  |  |  | |  |
| 14 | Guatemala, Chalatenango, Los Esesmiles | GUAT | 1 |  |  |  | |  |
| 15 | Guatemala, Huehuetenango, Santa Eulalia | GUAT | 1 |  |  |  | |  |
|  |  |  |  |  |  |  | |  |
|  | *Reithrodontomys sumichrasti*  GenBank acc. nos. AF211894–AF11923 [36] |  |  |  |  |  | |  |
| 1 | México, Hidalgo, Molango | nSMO | 2 |  |  |  | |  |
| 2 | México, Hidalgo, Zacoultipan | nSMO | 1 |  |  |  | |  |
| 3 | México, Hidalgo, Rancho La Mojonera | nSMO | 6 |  |  |  | |  |
| 4 | México, Hidalgo, Tenango de Doria | nSMO | 2 |  |  |  | |  |
| 5 | México, Veracruz, Zacualtapan | cSMO | 3 |  |  |  | |  |
| 6 | México, Veracruz, Xometla | cSMO | 2 |  |  |  | |  |
| 7 | México, Oaxaca, Puerto de la Soledad | sSMO | 4 |  |  |  | |  |
| 8 | México, Oaxaca, 27.5 km W Orizaba | sSMO | 2 |  |  |  | |  |
| 9 | México, Oaxaca, Suchixtepec | sSMO | 2 |  |  |  | |  |
| 10 | México, Guerrero, Omiltemi | WEST | 5 |  |  |  | |  |
| 11 | México, Guerrero, Filo de Caballo | WEST | 2 |  |  |  | |  |
| 12 | México, Michoacán, Mil Cumbres | WEST | 1 |  |  |  | |  |
| 13 | México, Michoacán, Opopeo | WEST | 3 |  |  |  | |  |
| 14 | México, Michoacán, Villa Escalante | WEST | 1 |  |  |  | |  |
| 15 | México, Chiapas, San Cristobal de las Casas | cCHIS | 4 |  |  |  | |  |
| 16 | México, Chiapas, Rayón | cCHIS | 1 |  |  |  | |  |
| 17 | México, Chiapas |  | 1 |  |  |  | |  |
| 18 | Costa Rica, San José, Cascajal de Coronado | CR | 1 |  |  |  | |  |
|  |  |  |  |  |  |  | |  |
|  | *Peromyscus aztecus* complex  GenBank acc. nos. U89965–U89983 [37] |  |  |  |  |  | |  |
|  | *Peromyscus winkelmanni* |  |  |  |  |  | |  |
| 1 | México, Michoacán, Dos Aguas | WEST | 2 |  |  |  | |  |
| 2 | México, Guerrero, Filo de Caballo | WEST | 1 |  |  |  | |  |
|  | *Peromyscus spicilegus* |  |  |  |  |  | |  |
| 3 | México, Michoacán, Uruapan | WEST | 2 |  |  |  | |  |
| 4 | México, Jalisco, Villa Carranza | WEST | 1 |  |  |  | |  |
|  | *Peromyscus hylocetes* |  |  |  |  |  | |  |
| 5 | México, Michoacán, Mil Cumbres | WEST | 1 |  |  |  | |  |
| 6 | México, Michoacán, Los Azufres | WEST | 1 |  |  |  | |  |
| 7 | México, Michoacán, Puerto Garnica | WEST | 2 |  |  |  | |  |
| 8 | México, Morelos, Huitzilac | WEST | 1 |  |  |  | |  |
|  | *Peromyscus a. aztecus* |  |  |  |  |  | |  |
| 9 | México, Veracruz, Huatusco | cSMO | 1 |  |  |  | |  |
| 10 | México, Veracruz, Teocelo | cSMO | 2 |  |  |  | |  |
|  | *Peromyscus a. oaxacensis* |  |  |  |  |  | |  |
| 11 | México, Oaxaca, Llano de las Flores | sSMO | 5 |  |  |  | |  |
| 12 | México, Guatemala, Zacapa, San Lorenzo | GUAT | 3 |  |  |  | |  |
|  | *Peromyscus a. evides* |  |  |  |  |  | |  |
| 13 | México, Oaxaca, Juquila | sSMO | 2 |  |  |  | |  |
| 14 | México, Oaxaca, Suchixtepec | sSMO | 1 |  |  |  | |  |
