# Supplementary material for: Comparative Phylogeographic Analyses Illustrate the Complex Evolutionary History of Threatened Cloud Forests of Northern Mesoamerica
Source: PLoS One. 2013 Feb 7;8(2):e56283. doi: 10.1371/journal.pone.0056283 (PMC3567015; doi:10.1371/journal.pone.0056283)
Supplement: Table S2 — Sample sizes, molecular markers, substitution models, substitution rates, and temporal calibrations used in this study. (DOC) [file pone.0056283.s002.doc]

**Table S2.** Sample sizes, molecular markers, substitution models, substitution rates, and temporal calibrations used in this study.

|  |  |  |  |  |  |
| --- | --- | --- | --- | --- | --- |
| **Taxon** | **No. ind.** | **Molecular markers** | **Substitution models** | **Substitution rates** | **Secondary and fossil calibrations** |
|  |  |  |  |  |  |
| **Plants** |  |  |  |  |  |
| *Podocarpus matudae* ^a, b^ | 174 | *trn*L-F, *psb*A-*trn*H | HKY+I | - | Ornelas *et al*. [3] |
| *Liquidambar styraciflua* ^a^ | 134 | *psb*A-*trn*H | HKY+G | - | Morris *et al*. [1] |
| *Palicourea padifolia* ^a, c^ | 122 | *rpl*32-*trn*L, *trn*S-*trn*G | HKY+G | - | Bremer & Eriksson [15] |
| *Moussonia deppeana* ^a^ | 193 | ITS, *rpl*32-*trn*L | GTR+I+G | - | Roalson *et al*. [17] |
| *Rhipsalis baccifera* ^a^, ^d^ | 158 | *rpl*32-*trn*L | HKY+I | - | Arakaki *et al*. [20] |
|  |  |  |  |  |  |
| **Birds** |  |  |  |  |  |
| *Campylopterus curvipennis* ^a, e^ | 162 | ATP6, ATP8 | HKY+G | 0.0125 s/s/l/My |  |
| *Amazilia cyanocephala* ^a, f^ | 133 | ATP6, ATP8 | HKY+G | 0.0125 s/s/l/My |  |
| *Lampornis amethystinus* ^a, g^ | 104 | CYTB, ND2 | GTR+I+G | 0.0125 s/s/l/My |  |
| *Lepidocolaptes affinis* ^h^ | 80 | CYTB, ND2 | GTR+G | - | Irestedt *et al*. [32] |
| *Buarremon brunneinucha* ^i^ | 48 | ATP6, ATP8 | GTR+I+G | 0.0125 s/s/l/My |  |
| *Basileuterus belli* ^a^ | 83 | ND2, ND5 | HKY+G | 0.0125 s/s/l/My |  |
| *Chlorospingus ophthalmicus* ^a, j^ | 67 | ATP6, ATP8 | GTR+I | 0.0125 s/s/l/My |  |
|  |  |  |  |  |  |
| **Rodents** |  |  |  |  |  |
| *Habromys “lophurus”* ^k^ | 31 | ND3, ND4 | HKY+I+G | - | Steppan *et al*. [35] |
| *Reithrodontomys sumichrasti* ^l, m^ | 30 | CYTB | GTR+I+G | - | Steppan *et al*. [35] |
| *Peromyscus “aztecus”* ^l^ | 18 | CYTB | GTR+I+G | - | Steppan *et al*. [35] |

Sequence sources (see full references in **Text S1**).

^a^ This study.

^b^ Ornelas *et al*. [3].

^c^ Gutiérrez-Rodríguez *et al*. [14].

^d^ Calvente *et al*. [18].

^e^ González *et al*. [26].

^f^ Rodríguez-Gómez *et al*. [28].

^g^ Cortés-Rodríguez *et* *al*. [27].

^h^ Arbeláez-Cortés *et al*. [31].

^i^ Navarro-Sigüenza *et al*. [30].

^j^ Bonaccorso *et al*. [29].

^k^ León-Paniagua *et al*. [33].

^l^ Sullivan *et al*. [35].

^m^ Sullivan *et al*. [36].
