# Supplementary material for: Comparative Phylogeographic Analyses Illustrate the Complex Evolutionary History of Threatened Cloud Forests of Northern Mesoamerica
Source: PLoS One. 2013 Feb 7;8(2):e56283. doi: 10.1371/journal.pone.0056283 (PMC3567015; doi:10.1371/journal.pone.0056283)
Supplement: Table S3 — Summary statistics for sequence divergence by species. (DOC) [file pone.0056283.s003.doc]

**Table S3**. Summary statistics for sequence divergence by species. Abbreviations as follow: sequence length (bp), number of individuals sampled (N), and percent (mean ± SD) genetic distances within (D*x*, D*y*) and between populations separated by geographic barriers corrected for intra-population polymorphism (D*_xy_*). See **Text S1** for sequence sources.

|  |  |  |  |  |  |  |
| --- | --- | --- | --- | --- | --- | --- |
| **Geographic barrier** | **Locus** | **bp** | **N**  ***x/y*** | **%D*x*** | **%D*y*** | **%D*xy*** |
| **Isthmus of Tehuantepec** |  |  |  |  |  |  |
| *Palicourea padifolia* | *trn*S-*trn*G, *rpl*32-*trn*L | 1393 | 105/17 | 0.055 (0.058) | 0.048 (0.062) | 0.199 (0.065) |
| *Rhipsalis baccifera* | *rpl*32-*trn*L | 1074 | 134/20 | 0.071 (0.119) | 0.075 (0.171) | 0.118 (0.154) |
| *Campylopterus curvipennis* | ATP6, ATP8 | 1407 | 138/21 | 0.282 (0.199) | 0.248 (0.158) | 3.047 (0.142) |
| *Amazilia cyanocephala* | ATP6, ATP8 | 1218 | 77/49 | 0.144 (0.098) | 0.232 (0.140) | 0.285 (0.129) |
| *Lampornis amethystinus* | CYTB, ND2 | 762 | 76/22 | 0.872 (0.340) | 0.502 (0.326) | 1.504 (0.344) |
| *Lepidocolaptes affinis* | CYTB, ND2 | 1869 | 45/25 | 0.225 (0.123) | 0.143 (0.090) | 0.253 (0.078) |
| *Basileuterus belli* | ND2, ND5 | 798 | 51/21 | 0.336 (0.201) | 0.867 (0.539) | 1.746 (0.221) |
| *Habromys “lophurus”* | ND3, ND4 | 1330 | 21/9 | 10.243 (5.709) | 2.668 (1.097) | 13.975 (1.928) |
| *Reithrodontomys sumichrasti* | CYTB | 1130 | 23/7 | 3.740 (2.288) | 1.090 (0.846) | 7.152 (0.674) |
| *Peromyscus “aztecus”* | CYTB | 664 | 20/3 | 5.849 (2.926) | 0 | 8.563 (0.597) |
| **Los Tuxtlas** |  |  |  |  |  |  |
| *Liquidambar styraciflua* | *psb*A-*trn*H | 382 | 125/9 | 0.087 (0.139) | 0 | 0.362 (0.117) |
| *Campylopterus curvipennis* | ATP6, ATP8 | 1407 | 126/12 | 0.226 (0.153) | 0.105 (0.087) | 0.615 (0.076) |
| *Buarremon brunneinucha* | ATP6, ATP8 | 800 | 15/2 | 0.251 | 0.496 (0.251) | 3.845 (0.193) |
| *Basileuterus belli* | ND2, ND5 | 798 | 13/5 | 0.249 (0.208) | 0.101 (0.130) | 0.564 (0.186) |
| *Chlorospingus ophthalmicus* | ATP6, ATP8 | 321 | 137/15 | 1.603 (2.842) | 2.919 (2.002) | 7.397 (1.577) |
| **Isthmus 2** |  |  |  |  |  |  |
| *Podocarpus matudae* | *psb*A-*trn*H, *trn*L-F | 1035 | 127/32 | 0.553 (0.467) | 0.294 (0.333) | 0.511 (0.369) |
| *Moussonia deppeana* | ITS, *rpl*32-*trn*L | 1226 | 133/60 | 0.545 (0.271) | 0.283 (0.156) | 0.546 (0.217) |
| *Chlorospingus ophthalmicus* | ATP6, ATP8 | 316 | 130/46 | 3.927 (2.900) | 3.386 (2.589) | 8.141 (1.892) |
| **Chiapas Central Depression** |  |  |  |  |  |  |
| *Lampornis amethystinus* | CYTB, ND2 | 762 | 16/6 | 0.859 (0.341) | 0.396 (0.229) | 0.960 (0.285) |
| *Basileuterus belli* | ND2, ND5 | 798 | 10/11 | 0.297 (0.185) | 0.087 (0.108) | 0.477 (0.079) |

Isthmus of Tehuantepec (east and west of the isthmus), Los Tuxtlas (isolated from the Sierra Madre Oriental), Chiapas Central Depression (populations in Chiapas separated by the Central Depression), Isthmus 2 (the Chiapas and Los Tuxtlas populations separated from populations west of the Isthmus of Tehuantepec).
