# Supplementary material for: Comparative Phylogeographic Analyses Illustrate the Complex Evolutionary History of Threatened Cloud Forests of Northern Mesoamerica
Source: PLoS One. 2013 Feb 7;8(2):e56283. doi: 10.1371/journal.pone.0056283 (PMC3567015; doi:10.1371/journal.pone.0056283)
Supplement: Text S1 — Details of nucleotide evolutionary models used, fossil and secondary calibrations, substitution rates and taxon sampling to divergence time estimation. (DOC) [file pone.0056283.s004.doc]

**Text S1.** Details of nucleotide evolutionary models used, fossil and secondary calibrations, substitution rates, topological constraints, prior distribution and parameter values input into BEAUti, and taxon sampling to divergence time estimation.

**Materials and Methods**

Divergence time estimation

Because the tree priors available in the current version of BEAST are not designed to model mixed inter- and intraspecific data, and because the potential problems associated with model parameter variance among heterogeneous datasets, the coalescent tree prior was used in all cases which appears to be a better fit when mixed datasets are predominantly intraspecific data [1]. In *Podocarpus matudae*, divergence time for the Podocarpaceae family using the *trn*L-F (483 bp) sequences of Sinclair *et al*. [2] (accession numbers: AY083071–AY083112) plus 14 samples of *P. matudae* haplotypes from Ornelas *et al*. [3] was estimated using *Agathis australis* (Araucariaceae) as a functional outgroup. We used the HKY+G substitution model based on the result of AIC from jModelTest v. 0.1.1 [4] for this analysis and an uncorrelated lognormal relaxed model selected in BEAST as the clock model. A Yule speciation model was used to model the tree prior. We constrained four genera to be monophyletic according to Sinclair *et al*. [2]: Podocarpaceae, *Phyllocladus*, *Podocarpus* including our *P. matudae* samples, *Dacrycarpus* and *Dacrydium*. For fossil calibration points we used a lognormal prior distribution with the offset adjusted to correspond to the fossil age (the minimum age of the node) and for secondary calibrations we used a normal prior distribution to produce a median age of exactly the secondary calibration and a standard deviation to include its 95% highest posterior density (HPD) intervals [5,6]. The divergence time between Araucariaceae and Podocarpaceae, 257 Ma (95% HPD 287–228, [7]), was used as secondary calibration to calibrate the root node (normal distribution, mean 257, SD 14.8, range 286–228 Ma). For the *Phyllocladus* crown group, the age of Jurassic fossil pollen of 195 Ma from New Zealand [8] was used (lognormal distribution, mean 0.0, SD 1.0, offset 195, range 202.1–195 Ma). For the *Dacrycarpus* crown group, the age of an Eocene fossil of 25 Ma from Australia [9] was used (lognormal distribution, mean 0.0, SD 1.0, offset 25, range 32–25 Ma). For the crown group of *Dacrydium*, an Oligocene (35 Ma) fossil [10] from Tasmania was used (lognormal distribution, mean 0.0 SD 1.0, offset 35, range 42–35 Ma). Lastly, we used a fossil [11] from the Eocene of North America for the *Podocarpus* crown group, with an age of 56 Ma (lognormal distribution, mean 0.0, SD 1.0, offset 56, range 63–56 Ma). A second estimation of divergence times was conducted using two cpDNA regions (*trn*L-F and *psb*A-*trn*H; 1057 bp) of all *P. matudae* samples from Ornelas *et al*. [3], 20 new samples (JX556873–JX556880, JX556900–JX556906), and *P. macrophyllus* as outgroup. We used the HKY+I model based on the result of AIC from jModelTest for this analysis and an uncorrelated lognormal relaxed model selected in BEAST as the clock model. A coalescent model assuming population constant size was used to model the tree prior. To calibrate the root, we used the results of the first divergence estimation of Podocarpaceae estimated 32.05 Ma, 95% HPD 47.09–17.12 (normal distribution, mean 32.05, SD 7.63, range of 47–17.1 Ma) divergence time for the *Podocarpus matudae* crown group.

In *Liquidambar styraciflua* (Altingiaceae), we estimated the divergence time to the Altingiaceae using the *psb*A*-trn*H (349 bp) sequences of Morris *et al*. [1] of *Liquidambar acalycina* (EU595860) *L. formosana* (EU595861), *L. orientalis* (EU595855), *Altingia chinensis* (EU595856), *Altingia excelsa* (EU595859), *Altingia obovata* (EU595862), *Altingia poilanei* (EU595858), *Altingia yunnanensis* (EU595857) and *Hamamelis virginiana* (EU595863) plus *L. styraciflua* haplotypes from USA populations (EF138708–EF138724) and our *psb*A*-trn*H sequence data of Mexican populations (JX556867–JX556872). We used the model of selection HKY+G based on the result of BIC from jModelTest for this analysis and an uncorrelated lognormal relaxed clock model selected in BEAST as the clock model. A coalescent model assuming population constant size was used to model the tree prior. We constrained the *Liquidambar* crown clade and the *L. styraciflua* crown clade based on Morris *et al*. [1] and our *psb*A*-trn*H haplotype network and Bayesian tree. *Microaltingia* (90 Ma) from the late Cretaceous of New Jersey [12] was used to calibrate the root node (lognormal distribution, mean 0.0, SD 1.0, offset 90, range 97–90 Ma). *Liquidambar changii* (15.6 Ma) from Middle Miocene of eastern Washington [13] was used to calibrate the clade containing *Liquidambar formosana*, *L. obovata* and *L. acalycina* (lognormal distribution, mean 0.0, SD 1.5, offset 15.6, range 34.5–15.6 Ma). For the *L. styraciflua* crown group, we set an age of 3 Ma on the basis of fossil material [1] from the Citronelle formation of southern Alabama (lognormal distribution, mean 0.0, SD 1.0, offset 3, range 10.1–3.1 Ma).

The *trn*S*-trn*G and *rpl*32-*trn*L (1393 bp) sequences (JF891318–JF891381) of Gutiérrez-Rodríguez *et al*. [14] were used to estimate the time to the most common recent ancestor (tMRCA) of the resulting two genetic groups of *Palicourea padifolia* (Rubiaceae) in Mexico. Samples from Costa Rica, Panama and Colombia were included; and *Faramea occidentalis* (JF891347, JF891379) and two species of *Psychotria* (JF891348–JF891380, JF891349–JF891380) were used as outgroups. The analysis was performed using the HKY+G model based on the result of AIC from jModelTest for this analysis and an uncorrelated lognormal relaxed clock model selected in BEAST as the clock model. A coalescent model assuming population constant size was used to model the tree prior. We constrained all the sequences, except for *F. occidentalis*, as monophyletic based on the results of Bremer & Ericson [15], and samples from either side of the Isthmus of Tehuantepec according to Gutiérrez-Rodríguez *et al*. [14]. The age of the subfamily Rubioideae crown node estimated at 77.9 Ma (95% HPD 90.7–65.3 Ma; [15]) using the *Faramea* pollen fossil (37 Ma) from the Upper Eocene found at the Gatuncillo Formation near Alcalde Diaz in Panama [16] was used as secondary calibration to calibrate the root node of the tree (normal distribution, mean 77.9, SD 6.2, range of 90–65.7 Ma). The node age of the Psychotrieae crown group of 35.6 Ma 95% HPD 46.9–25.5 Ma (normal distribution, mean 35.6, SD 5.2, range of 45.79–25.5 Ma) was used as a prior for the tMRCA of the clade containing *Palicourea* and *Psychotria* sequences.

For the *Moussonia deppeana* (Gesneriaceae) data (JX847822–JX847850, JX847851–JX847883), we estimated the divergence time for the Gloxinieae tribe representatives using the ITS (500 bp) sequences of *Achimenes* (AY047065, AY047066, AY047067)*,* *Kohleria* (AY702371, AY702372, AY702373, AY702374, AY702375, AY047075, AY702377, AY047076, AY702379), *Niphaea* (AY047064), and *Moussonia* (AY702383, AY702384, AY047068) of Roalson *et al*. [17] plus haplotypes from our *M. deppeana* ITS data. We used the HKY+I model based on the result of BIC from jModelTest for this analysis and an uncorrelated lognormal relaxed clock model selected in BEAST as the clock model. A Yule speciation model was used to model the tree prior. Topological constraints were imposed in those nodes where a particular resolution was needed for subsequent calibrations. Thus, we constrained *Achimenes*, *Kohleria*, *Moussonia* and five groups (Sierra Madre Oriental, Los Tuxtlas, Chiapas and Guatemala, Sierra de Manantlán, Sierra de Miahuatlán) to be monophyletic based on Roalson *et al*. [17], and the ITS ribotype network and Bayesian tree. For temporal calibration of the root node of the tree, we used the divergence time between Gesnerieae and Gloxinieae (26 Ma, 95% HPD 22.47–29.47 Ma) of node 32 [17] as secondary calibration (normal distribution, mean 26, SD 1.9, range of 29.72–22.28 Ma). For the *M. deppeana* crown group, the divergence between *M.* *septentrionalis* and the *Niphaea-Smithiantha-Eucodonia* clade of 17 Ma (95% HPD 18.84–14.48 Ma; node 13 of [17]) was used as secondary calibration (normal distribution, mean 17, SD 1.1, range 19.16–14.48 Ma). For the *Kohleria* crown group, the age of the *Kohleria* crown clade 6.8 Ma (95% HPD 8.71–4.91; node 4 of [17]) was used as secondary calibration (normal distribution, mean 6.8, SD 1.0, range 8.76–4.84 Ma). The second estimation of divergence times was conducted for the *M. deppeana* complex based on the combined ITS/*rpl*32-*trn*L (500 + 685 bp) tree based on Bayesian inference. We used the GTR+I+G model of sequence evolution from jModelTest for this analysis and an uncorrelated lognormal relaxed clock model selected in BEAST as the clock model. A coalescent model assuming population constant size was used to model the tree prior. To calibrate the root, we used the results of the first divergence estimation of Gesneriaceae using the estimated 6.52 Ma 95% HPD 11–2.88 Ma (normal distribution, mean 6.52, SD 2.3, range 11.03–2.01 Ma) divergence time for the *M. deppeana* crown group. We used the same constraints for the *M. deppeana* crown group described above.

In *Rhipsalis baccifera* (Cactaceae), we estimated the divergence time to the Cactaceae using the *rpl*32-*trn*L (1425 bp) sequences of Calvente *et al*. [18] of *Rhipsalis cereoides* (HQ727859), *R. crispata (*HQ727850), *R. elliptica* (HQ727849), *R. micrantha* (HQ727855), *R. olivifera* (HQ727860), *R. pachyptera (*HQ727851), *R. russellii* (HQ727865), *R. neves-armondii* (HQ727856), *R. puniceodiscus* (HQ727868), *R. dissimilis* (HQ727869), *R. floccosa* (HQ727867), *R. paradoxa* (HQ727861), *R. trigona* (HQ727857), *R. baccifera* (HQ727863), *R. lindbergiana* (HQ727874), *R. mesembryanthemoides* (HQ727858), *R. teres* (HQ727873), *R. clavata* (HQ727872), *R. pulcra* (HQ727854), *R. cereuscula* (HQ727882), *R. pilocarpa* (HQ727864), *Hatiora salicornioides* (HQ727862), *H. cilindrica* (HQ727871), *Lepismium cruciforme* (HQ727866), *L. lumbricoides* (HQ727877), *L. houlletianum* (HQ727875), *L. warmingianum* (HQ727878), *Schlumbergera truncata* (HQ727876), *S. russelliana* (HQ727853), *S. opuntioides* (HQ727880), *S. orssichiana* (HQ727852), *Pereskia bahiensis* (HQ727881), *Pfeiffera ianthothele* (HQ727883) and *Epiphyllum phyllanthus* (HQ727886) plus samples from our *Rhipsalis baccifera* *rpl*32-*trn*L data (JX556881–JX556899). We used the HKY+I model based on the result of BIC from jModelTest for this analysis and an uncorrelated lognormal relaxed clock model selected in BEAST as the clock model. A coalescent model assuming population constant size was used to model the tree prior. We constrained Cactoideae, Rhipsalideae, *Rhipsalis*, and *R. baccifera* to be monophyletic based on Calvente *et al*. [18], Edwards *et al*. [19], Arakaki *et al*. [20], and our *rpl*32-*trn*L network and Bayesian tree. For temporal calibration of the root node of the tree, we used as secondary calibration the age estimated for the Cactaceae estimated at 35 Ma (95% HPD 41–29; normal distribution, mean 35, SD 3.0, range 40.88–29.12 Ma) based on Arakaki *et al*. [20]. For the Cactoideae, Rhipsalideae, and *Rhipsalis* crown groups, the ages of 24.48 Ma (95% HPD 28.75–20.69 Ma; normal distribution, mean 24.48, SD 2.2, range 28.79–20.17 Ma), 16 Ma (95% HPD 18.85–13.57 Ma; normal distribution, mean 16, SD 1.5, range 18.94–13.06 Ma), and 10 Ma (95% HPD 11.78–8.47 Ma; normal distribution, mean 10, SD 0.91, range 11.78–8.21 Ma) were assigned as secondary calibrations, respectively, according to divergence time estimates by Arakaki *et al*. [20].

For all bird species, tMRCA was estimated for the resulting clades using a Bayesian MCMC sampling approach with BEAST. We used models of sequence evolution (**Table S2**) from jModelTest for this analysis and an uncorrelated lognormal relaxed clock model selected in BEAST as the clock model. A coalescent model assuming population constant size was used to model the tree prior, with other priors set to default values. No topological constraints were used allowing topological uncertainty to be taken into account. In the absence of appropriate internal calibration points for many groups of birds, the 2% divergence-per-My clock calibration has been widely used. In a recent study, Weir & Schluter [21] cross-validated 90 avian clock calibrations for CYTB obtained from fossil records and biogeographic events, demonstrating support for the 2% rule across taxonomic orders. However, the degree of heterogeneity of molecular evolution rates across lineages and genetic loci could confound the accuracy of divergence time estimates, making the use of the 2% rule controversial [22–24]. Given that ATP6, ATP8 and ND2 evolve at approximately 1.25 times the rate of CYTB, we applied a rate of 2.5% substitutions/site per million years (0.0125 substitutions/site/lineage/million years) to *Campylopterus curvipennis*, *Lampornis amethystinus*, *Amazilia cyanocephala* (Trochilidae), *Basileuterus belli* (Parulidae), *Chlorospingus ophthalmicus* and *Buarremon brunneinucha* (Emberizidae) according to Smith and Klicka [25]. We estimated the divergence time to the *C. curvipennis* species complex using the ATP6 and ATP8 sequences (875 bp) of González *et al*. [26] of *Campylopterus rufus* (HQ380754), *C.* *largipennis* (HQ380755), *C. hemileucurus* (HQ380753) and *C. villaviscencio* (JX847799) plus 162 individuals of the *C. curvipennis* species complex (HQ380727–HQ380752). The divergence time estimation of *L. amethystinus* was estimated using 69 sequences of ND2 (354 bp) and CYTB (490 bp) (EU543284–EU543433) of Cortés-Rodríguez *et al*. [27] plus 35 new samples (JX847800–JX847807, JX847808–JX847821) of *L. amethystinus*, and *Lampornis clemenciae* (EU543354, EU543429), *L. sybillae* (EU543356, EU543431), *L. viridipallens* (EU543355, EU543430), *L. calolaemus* (EU543357, EU543432), *Lamprolaima rhami* (EU543358, EU543433) and *Hylocharis leucotis* (EU418759, DQ196556) as outgroups. We estimated the divergence time to the *A. cyanocephala* species complex using the ATP6 and ATP8 sequences (770 bp) of Rodríguez-Gómez *et al*. [28] of *Amazilia beryllina* (JX675221), *A.* *violiceps* (JX675222) and *A. viridifrons* (JX675223) plus 133 individuals of the *A. cyanocephala* species complex (JX050059–JX050109). Divergence time to the *Basileuterus belli* species complex was estimated using ND2 (362 bp) and ND5 (335 bp) sequences of the outgroups *Basileuterus rufifrons*, *B. culicivorus*, *B. fulvicauda*, *B.* *rivularis*, *B. coronatus*, *B. luteoviridis*, *B. nigrocristatus*, *B. leucoblepharus*, *B. flaveolus* and *B. tristriatus* plus 83 individuals of the *B. belli* species complex (JX626333–JX626402). The divergence time estimation of *C. ophthalmicus* was estimated using 67 ATP6 (527 bp) and ATP8 (168 bp) sequences (EU594945–EU595009) of Bonaccorso *et al*. [29] of *C. ophthalmicus*, and *Chlorospingus canigularis* (AF447322), *Aimophila cassinii* (AF447312), *Junco hyemalis* (AF447338), *Atlapetes schistaceus* (AF447313) and *Calamospiza melanocorys* (AF447316) as outgroups. The divergence time estimation of *Buarremon brunneinucha* was estimated using 48 ATP6 and ATP8 (801 bp) sequences (EU594945–EU595009) of Navarro-Sigüenza *et al*. [30] of *B. brunneinucha*, and *Atlapetes pileatus* (EU364969, EU364970), *Junco phaeonotus* (AF468825), *Junco hyemalis* (AF447338) and *Calamospiza melanocorys* (AF447316) as outgroups. Lastly, the divergence time estimation of *Lepidocolaptes affinis* was estimated using 80 ND2 (903 bp) and CYTB (966 bp) sequences (HQ014479–HQ014562) of Arbeláez-Cortés *et al*. [31] of *L. affinis*, and *Lepidocolaptes leucogaster* (GU215191, GU215382), *L. lachrymiger* (GQ906720, GU215190), *L. angustirostris* (AY089838, AY089811), *Sittasomus griseicapillus* (GU215383, GU215197) and *Xyphorhynchus flavigaster* (AY089871, AY089799) from the GenBank as outgroups. For temporal calibration of the root node of the tree, we used as secondary calibration the age estimated for the *Xenops*/Dendrocolaptidae estimated at 27.7 Ma (95% HPD 32.6–23.49 Ma; normal distribution, mean 27.7, SD 2.5, range 32.6–22.8 Ma) based on Irestedt *et al*. [32].

For all rodent species, tMRCA was estimated for the resulting clades using a Bayesian MCMC sampling approach with BEAST. We used models of sequence evolution (**Table S2**) from jModelTest for this analysis and an uncorrelated lognormal relaxed clock model selected in BEAST as the clock model. A coalescent model assuming population constant size was used to model the tree prior, with other priors set to default values. No topological constraints were used allowing topological uncertainty to be taken into account. In the case of *Habromys* rodents, we used 31 ND3 and ND4 (1331 bp) sequences (DQ793090–DQ793118) of León-Paniagua *et al*. [33] of the *Habromys “lophurus”* species complex (*simulatus*, *delicatus*, *schmidlyi*, *chinanteco*, *lepturus*, *ixtlani* and *lophurus*), and *Peromyscus boylli* (U83864), *P. slevini* (PSU40248), *P. melanotis* (PMU40247), *P. maniculatus* (PMU40247), *P. polynotus* (PMU40247), *P. leucotis* (PLU40252), *P. eremicus* (PEU83861), *P. mexicanus* (U83862, PMU83862), *Osgodomys banderanus* (OBU83860), *Onychomys leucogaster* (OLU83858), *Podomys floridanus* (PFU83865), *Baiomys taylori* (BTU83829) and *Neotoma floridana* (NFU83827) from the GenBank as outgroups. For temporal calibration of the root node of the tree, we used as secondary calibration the age estimated for Neotominae estimated at 10.9 Ma (95% HPD 11–7.7 Ma; normal distribution, mean 10.9, SD 1.6, range 14.04–7.76 Ma) based on a fossil-based (*Copemys* *russelli*, 14.8 Ma; [34]) divergence dates in Muroid rodents by Steppan *et al*. [35]. Thirty CYTB (1130 bp) sequences of *Reithrodontomys sumichrasti* (AF211894–AF211923) were examined of Sullivan *et al*. [36]. In addition, *Reithrodontomys megalotis* (AY859468), *R.* *microdon* (AY859454), *Peromyscus grandis* (GQ461925), *P. guatemalensis* (GQ461935), *P.* *mexicanus* (EF989994), *P. zarhynchus* (AY195800), *P. mayensis* (EF989987), *P. melanocarpus* (EF028173), *P. magalopus* (DQ000475), *P. perfulvus* (DQ000474), and *Mus musculus* (AF520635, AF520634, AY057804) from the GenBank were included as outgroup taxa. For temporal calibration of the root node of the tree, we used as secondary calibration the murid group split from the cricetid group estimated at 24.2 Ma (95% HPD 24.7–22 Ma; normal distribution, mean 24.2, SD 0.9, range 25.96–22.44 Ma) and the split between *Peromyscus* and *Reithrodontomys* at 10.9 Ma (95% HPD 11–7.7 Ma; normal distribution, mean 10.9, SD 1.6, range 14.04–7.76 Ma) based on divergence dates in Muroid rodents by Steppan *et al*. [35]. Lastly, divergence estimates of the *Peromyscus “aztecus”* group CYTB (719 bp) data included 18 samples of Sullivan *et al*. [37]. In addition, *P. boylii* (PBU89965), *Reithrodontomys megalotis* (AY859468), *R.* *microdon* (AY859454), *Peromyscus grandis* (GQ461925), *P. guatemalensis* (GQ461935), *P.* *mexicanus* (EF989994), *P. zarhynchus* (AY195800), *P. mayensis* (EF989987), *P. melanocarpus* (EF028173), *P. magalopus* (DQ000475), *P. perfulvus* (DQ000474), and *Mus musculus* (AF520635, AF520634, AY057804) from the GenBank were included as outgroup taxa. The same calibration approach used for *R. sumichrasti* was implemented here.

**References**

1. Morris AB, Ickert-Bond SM, Brunson B, Soltis DE, Soltis PS (2008) Phylogeographical structure and temporal complexity in American sweetgum (*Liquidambar styraciflua*; Altingiaceae). Mol Ecol 17: 3889–3900.
2. Sinclair WT, Mill RR, Gardner MF, Woltz P, Jaffré T, Preston J, Hollingsworth ML, Ponge A, Möller M (2002) Evolutionary relationships of the New Caledonian heterotrophic conifer, *Parasitaxus usta* (Podocarpaceae), inferred from chloroplast *trn*L-F intron/spacer and nuclear rDNA ITS2 sequences. Pl Syst Evol 233: 79–104.
3. Ornelas JF, Ruiz-Sánchez E, Sosa V (2010) Phylogeography of *Podocarpus matudae* (Podocarpaceae): pre-Quaternary relicts in northern Mesoamerican cloud forests. J Biogeogr 37: 2384–2396.
4. Posada D (2008) jModelTest: Phylogenetic Model Averaging. Mol Biol Evol 25: 1253–1256.
5. Ho SYW (2007) Calibrating molecular estimates of substitution rates and divergence time in birds. J Avian Biol 38: 409–414.
6. Ho SYW, Phillips MJ (2009) Accounting for calibration uncertainty in phylogenetic estimation of evolutionary divergence times. Syst Biol 58: 367–380.
7. Renner SS (2009) Gymnosperms. The timetree of life (ed. by S.B. Hedges and S. Kumar), pp. 157–160. Oxford University Press, Oxford, UK.
8. Wagstaff SJ (2004) Evolution and biogeography of the austral genus *Phyllocladus* (Podocarpaceae). J Biogeogr 31: 1569–1577.
9. Greenwood DR (1987) Early Tertiary Podocarpaceae -megafossils from the Eocene Anglesea locality, Victoria, Australia. Aust J Bot 35: 111–134.
10. Wells PM, Hill RS (1989) Fossil imbricate-leaved Podocarpaceae from Tertiary sediments in Tasmania. Aust J Bot 2: 387–423.
11. Dilcher DL (1969) *Podocarpus* from the Eocene of North America. Science 164: 299–301.
12. Zhou ZK, Crepet WL, Nixon KC (2001) The earliest fossil evidence of the Hamamelidaceae: Late Cretaceous (Turonian) inflorescences and fruits of Altingioideae. Am J Bot 88: 753–766.
13. Pigg KB, Ickert-Bond SM, Wen J (2004) Anatomically preserved *Liquidambar* (Altingiaceae) from the Middle Miocene of Yakima Canyon, Washington State, USA and its biogeographic implications. Am J Bot 91: 499–509.
14. Gutiérrez-Rodríguez C, Ornelas JF, Rodríguez-Gómez F (2011) Chloroplast DNA phylogeography of a distylous shrub (*Palicourea padifolia*, Rubiaceae) reveals past fragmentation and demographic expansion in Mexican cloud forests. Mol Phylogenet Evol 61: 603–615.
15. Bremer B, Eriksson T (2009) Timetree of Rubiaceae –phylogeny and dating the family, subfamilies and tribes. Int J Pl Sci 170: 766–793.
16. Graham A (1985) Studies in Neotropical paleobotany. IV. The Eocene communities of Panama. Ann Miss Bot Gard 72: 504–534.
17. Roalson EH, Skog LE, Zimmer EA (2008) Untangling Gloxinieae (Gesneriaceae). II. Reconstructing biogeographic patterns and estimating divergence times among New World continental and island lineages. Syst Bot 33: 159–175.
18. Calvente AM, Zappi DC, Forest F, Lohmann LG (2011) Molecular phylogeny of tribe Rhipsalideae (Cactaceae) and taxonomic implications for *Schlumbergera* and *Hatiora*. Mol Phylogenet Evol 58: 456–468.
19. Edwards EJ, Nyffeler R, Donoghue MJ (2005) Basal cactus phylogeny: Implications of *Pereskia* (Cactaceae) paraphyly for the transition to the cactus life form. Am J Bot 92: 1177–1188.
20. Arakaki M, Christin P-C, Nyffeler R, Lendel A, Eggli U, Ogburn RM, Spriggs E, Moore MJ, Edwards EJ (2011) Contemporaneous and recent radiations of the world’s major succulent plant lineages. Proc Natl Acad Sci USA 108: 8379–8384.
21. Weir JT, Schluter D (2008) Calibrating the avian molecular clock. Mol Ecol 17: 2321–2328.
22. Arbogast BS, Edwards SV, Wakeley J, Beerli P, Slowinsli JB (2002) Estimating divergence times from molecular data on phylogenetic and population genetic timescales. Annu Rev Ecol Syst 33: 707–40.
23. García-Moreno J (2004) Is there a universal mtDNA clock for birds? J Avian Biol 35: 465–468.
24. Lovette IJ (2004) Mitochondrial dating and mixed support for the “2% rule% in birds. Auk 121: 1–6.
25. Smith BT, Klicka J (2010) The profound influence of the Late Pliocene Panamanian uplift on the exchange, diversification, and distribution of New World birds. Ecography 33: 333–342.
26. González C, Ornelas JF, Gutiérrez-Rodríguez C (2011) Selection and geographic isolation influence hummingbird speciation: genetic, acoustic and morphological divergence in the wedge-tailed sabrewing (*Campylopterus curvipennis*). BMC Evol Biol 11: 38.
27. Cortés-Rodríguez N, Hernández-Baños BE, Navarro-Sigüenza AG, Peterson AT, García-Moreno J (2008) Phylogeography and population genetics of the Amethyst-throated Hummingbird (*Lampornis* *amethystinus*). Mol Phylogenet Evol 48: 1–11.
28. Rodríguez-Gómez F, Gutiérrez-Rodríguez C, Ornelas JF (2013) Genetic, phenotypic and ecological divergence with gene flow at the Isthmus of Tehuantepec: the case of the Azure-crowned Hummingbird (*Amazilia cyanocephala*). J Biogeogr 00: 00–00.
29. Bonaccorso E, Navarro-Sigüenza AG, Sánchez-González LA, Peterson AT, García-Moreno J (2008) Genetic differentiation of the *Chlorospingus ophthalmicus* complex in Mexico and Central America. J Avian Biol 39: 311–321.
30. Navarro-Sigüenza AG, Peterson AT, Nyári A, García-Deras GM, García-Moreno J (2008) Phylogeography of the *Buarremon* brush-finch complex (Aves, Emberizidae) in Mesoamerica. Mol Phylogenet Evol 47: 21–35.
31. Arbeláez-Cortés E, Nyári Á, Navarro-Sigüenza AG (2010) The differential effects of lowlands on the phylogeographic pattern of a Mesoamerican montane species (*Lepidocolaptes* *affinis*: Lepidocolaptidae). Mol Phylogenet Evol 57: 658–668.
32. Irestedt M, Fejdlsa J, Dalén L, Ericson PPG (2009) Convergent evolution, habitat shifts and variable diversification rates in the ovenbird-woodcreeper family (Furnariidae). BMC Evol Biol 9: 268.
33. León-Paniagua L, Navarro-Sigüenza AG, Hernández-Baños BE, Morales JC (2007) Diversification of the arboreal mice of the genus *Habromys* (Rodentia: Cricetidae: Neotominae) in the Mesoamerican highlands. Mol Phylogenet Evol 42: 653–664.
34. Woodburne MO, Tedford RH, Swisher CC (1990) Lithostratigraphy, biostrtatigraphy, and geochronology of the Barstow Formation, Mojave desert, southern California. GSA Bull 102: 459–477.
35. Steppan SJ, Adkins RM, Anderson J (2006) Phylogeny and divergence-date estimates of rapid radiations in Muroid rodents based on multiple nuclear genes. Syst Biol 53: 533–553.
36. Sullivan J, Arellano E, Rogers DS (2000) Comparative phylogeography of Mesoamerican highland rodents: concerted versus independent response to past climatic fluctuations. Am Nat 155: 755–768.
37. Sullivan J, Markert JA, Kilpatrick CW (1997) Phylogeography and molecular systematics of the *Peromyscus aztecus* species group (Rodentia: Muridae) inferred using parsimony and likelihood. Syst Biol 46: 426–440.
